# Supplementary material for: An integrated risk and epidemiological model to estimate risk-stratified COVID-19 outcomes for Los Angeles County: March 1, 2020—March 1, 2021
Source: PLoS One. 2021 Jun 24;16(6):e0253549. doi: 10.1371/journal.pone.0253549 (PMC8224896; doi:10.1371/journal.pone.0253549)
Supplement: S1 Appendix — (PDF) [file pone.0253549.s001.pdf]

S1 Appendix

An integrated risk and epidemiological model to estimate risk-stratified COVID-19 outcomes for Los Angeles County: March 1, 2020 - March 1, 2021

Abigail L. Horn[1], Lai Jiang[1], Faith Washburn[2], Emil Hvitfeldt[1], Kayla de la Haye[1], William Nicholas[2], Paul Simon[2], Maryann Pentz[1], Wendy Cozen[1], Neeraj Sood[3,4], & David V. Conti[1]

[1] Department of Preventive Medicine
Keck School of Medicine
University of Southern California

[2] Los Angeles County Department of Public Health
313 N Figueroa St, Los Angeles, CA 90012

[3] Sol Price School of Public Policy
University of Southern California

[4] Schaeffer Center for Health Policy and Economics
University of Southern California

Contents

I SEIR+RISK Stochastic Epidemic Model 1

1 Epidemic model variables and parameters 1

1.1 State variables . . . 1

1.2 Fixed parameters (Transition times) . . . 1

1.3 Estimated parameters (transition branching probabilities) . . . 1

1.4 Deterministic differential equation model . . . 2

1.5 Stochastic dynamical state model . . . 2

Counting processes for transitions between states • System of equations for state variables and advancing the model • Cumulative and daily counts

1.6 Solving for the basic reproduction number, R_0 . . . 4

2 Parameter Estimation 5

2.1 Specifying prior parameter distributions . . . 6

Basic Reproduction Number, R_t • Time-varying Reproduction Number after Interventions, R_t • Fraction of observed infections r_t • Probabilities of disease stage progression • Starting time, t_0

2.2 Variable observations used for parameter estimation . . . 8

Count variables used in model estimation

2.3 Running the model . . . 9

Uncertainty quantification • Model implementation

2.4 Calculating population-wide CFR_t and IFR_t . . . 9

3 Epidemic model results 9

3.1 Estimated timeseries of numbers in infection states . . . 9

3.2 Parameter estimates . . . 9

3.3 Posterior parameter densities . . . 15

II Risk Model 18

4 Modeled risk factors 18

5 Step 2: Conditional RR for BMI, smoking, and comorbidities 19

5.1 Marginal relative risks between risk factors from published literature . . . 19

5.2 Correlation structure between risk factors . . . 19

5.3 Using JAM to estimate conditional RR . . . 19

6 Step 3: Prevalence of each risk profile in the infected population 19

6.1 Frequency of risk profiles in overall LAC population . . . 20

Data sources • Estimation

6.2 Frequency of risk profiles in infected population . . . 20

Profile design matrix • Calculation

7 Step 4: Risk-profile-stratified probabilities of disease stage progression 21

7.1 Overview . . . 21

7.2 Inputs . . . 21

Logit transformed population-average probabilities of each stage of disease $\hat{\alpha}_t$, $\hat{\kappa}_t$, $\hat{\delta}_t$ • Vector of conditional log risk estimates for model m, $\underline{\hat{\psi}}_m^{Cond}$ • Frequencies of each incoming population $\mathbf{f}_{t,q,in}$ • Mean-centered design matrix $\mathbf{X}_m$, and risk profile design matrix $\mathbf{R}$

7.3 Calculating risk-stratified probabilities . . . 22

8 Step 5: Conditional RR for age 22

9 Step 6: Risk-profile-stratified $CFR_{q,t}$ and $IFR_{q,t}$ 23

10 Results for risk-profile-stratified estimates 23

10.1 Comparison between observed and estimated frequency of each age group in deceased population (for Step 5) . . . 23

10.2 Profile-stratified probabilities of severe illness and death for LAC . . . 23

10.3 CFR(t) and IFR(t) over all risk profiles . . . 28

Part I
SEIR+RISK Stochastic Epidemic Model

1 Epidemic model variables and parameters

1.1 State variables

Variable	Description
P	Total population size
S	Susceptible population
E	Exposed not yet infectious
A	Infected, unobserved
I	Infected, observed
H	In Hospital
Q	In ICU
D	Dead
R	Recovered/removed

Table 1. State variables.

1.2 Fixed parameters (Transition times)

Parameter	Description	Value	Source
d_{EI}	days between exposure and infectivity (incubation period)	5 days	[1]
d_{IH}	days between symptom onset and hospitalization (if required)	10 days	[2]
d_{IR}	days between symptom onset and recovery (if not hospitalized)	7 days	[2]
d_{HQ}	days between hospitalization and ICU (if required)	1 day	[3]
d_{HR}	days between hospitalization and recovery (if ICU not required)	12 days	[4]
d_{QD}	days between ICU and fatality	8 days	[4]
d_{QR}	days between ICU and recovery	7 days	[4]

Table 2. Transition time parameters, values, and sources. These parameters are modeled as fixed values.

1.3 Estimated parameters (transition branching probabilities)

Parameter	Description
R_t	Basic reproductive number
β_t	Time-varying infectivity rate
r_t	Time-varying proportion of infections that are detected and reported out of all infections
α_t	Time-varying probability infected (I) patient requires hospitalization (H)
κ_t	Time-varying probability hospitalized (H) patient requires ICU (Q)
δ_t	Time-varying probability patient in ICU (Q) dies (D)

Table 3. Transition branching probabilities. These parameters are estimated.

1.4 Deterministic differential equation model

The "skeleton" [5] of the compartmental model is described by the following set of coupled ordinary differential equations (ODE) describing the transitions of individuals between the 8 compartments across time:

$$dS/dt = -\beta_t S(I+A) \tag{1}$$

$$dE/dt = \beta_t S(I+A) - \frac{1}{d_{EI}}E \tag{2}$$

$$dA/dt = \frac{1-r_t}{d_{EI}}E - \frac{1}{d_{IR}}A \tag{3}$$

$$dI/dt = \frac{r_t}{d_{EI}}E - (\frac{\alpha_t}{d_{IH}} + \frac{1-\alpha_t}{d_{IR}})I \tag{4}$$

$$dH/dt = \frac{\alpha_t}{d_{IH}}I - (\frac{\kappa_t}{d_{HQ}} + \frac{1-\kappa_t}{d_{HR}})H \tag{5}$$

$$dQ/dt = \frac{\kappa_t}{d_{HQ}}H - (\frac{\delta_t}{d_{QD}} + \frac{1-\delta_t}{d_{QR}})Q \tag{6}$$

$$dD/dt = \frac{\delta_t}{d_{QD}}Q \tag{7}$$

$$dR/dt = \frac{1-\alpha_t}{d_{IR}}I + \frac{1-\kappa_t}{d_{HR}}H + \frac{1-\delta_t}{d_{QR}}Q + \frac{1}{d_{IR}}A \tag{8}$$

With the total population size:

$$P = S+E+A+I+H+Q+D+R \tag{9}$$

1.5 Stochastic dynamical state model

We develop a stochastic discrete-time model of the dynamical state system, which provides an effective framework for parameter estimation and for generating confidence bounds reflecting stochasticity of the disease process in model projections.

We model the number of individuals leaving any of the classes by all available routes over a particular time interval by a set of coupled multinomial counting processes, $N_{X_i \to X_j}(t)$ for all transition pairs (i, j), with random transition rates. Transitions of individuals from one to the next stage of the disease are seen as stochastic movements between the corresponding population compartments at transition rates with random fluctuations. At each period an individual either stays or moves on to the next compartment. It has been proven that in the limit as the time interval $\Delta t \to 0$, the following discrete-time approximation process fulfills the properties defining a continuous time Markov process [6]. We do not model this continuous-time Markov process explicitly and operate only with its discrete approximation.

In general in this process, the random variable for the number of individuals leaving class X_i and going to X_j and X_k over time interval $[t, t+\Delta t)$, $(\Delta N_{X_i \to X_j}(t), \Delta N_{X_i \to X_k}(t))$, has the multinomial distribution

$$(\Delta N_{X_i \to X_j}(t), \Delta N_{X_i \to X_k}(t)) \sim \text{Multinomial}(X_i(t); p_{X_i \to X_j}(t), p_{X_i \to X_k}(t)),$$

for states X_j and X_k reachable from state X_i. The multinomial distributions result from summation over individual independent and identical Bernoulli trials for all members of each compartment [7, 8]. If there is only one possible transition direction out of X_i, then the distribution above is given by a binomial distribution.

The amount of time spent in a compartment is described by a Poisson process. The time length that an individual spends in a compartment is thus exponentially distributed with some compartment-specific rate $\lambda_i(t) = \lambda_{ij}(t) + \lambda_{ik}(t)$, where $\lambda_{ij}(t)$ and $\lambda_{ik}(t)$ are the rates to go from X_i to X_j and X_k, respectively. The probability of extending the stay by a further period of length Δt is $\exp(\lambda_i(t)\Delta t)$ and the probability of leaving is $1 - \exp(\lambda_i(t)\Delta t)$ [7]. Given there is a transition out of X_i, the conditional probability it is to X_j is $\frac{\lambda_{ij}}{\lambda_{ij}(t)+\lambda_{ik}(t)}$. Therefore the probability of leaving X_i and going to X_j in $[t, t+\Delta t)$, $p_{X_i \to X_j}$, is found as

$$p_{X_i \to X_j} = (1 - \exp(-(\lambda_{ij}(t) + \lambda_{ij}(t))\Delta t)) \frac{\lambda_{ij}}{\lambda_{ij}(t) + \lambda_{ik}(t)}.$$

In the following we proceed to define the increments over $[t, t+\Delta t)$ of all counting process, $\Delta N_{X_i \to X_j}(t)$, defining the transitions between all states in the process.

1.5.1 *Counting processes for transitions between states*

New latent infections (S to E transitions) New infections occur from direct or indirect interactions between susceptible (S) and infected (I or A) individuals, and arise first in the exposed (E) compartment. The rate at which susceptible individuals become infected is described by the *force of infection*, Λ, which combines the rate of infections per day per infected individual (β_t) across all infected individuals by multiplying with the overall fraction of infected individuals at time t, $\frac{I(t)+A(t)}{P}$, where P is equal to the total population size; $\Lambda(t) = \beta_t \frac{I(t)+A(t)}{P}$.

We assume that the corresponding probability at time t that a susceptible individual becomes infected and leaves the E compartment in one time unit Δt is equal to $p_{S\to}(t) = 1 - e^{-\beta_t \frac{I(t)+A(t)}{P}\Delta t}$.

Then, the overall number of individuals becoming infected and moving from the S to the E compartment in one time step, $\Delta N_{S\to E}$, is distributed as a binomial distribution with one draw for each of S individuals, each with probability $p_{S\to E}(t)$, i.e.

$$\Delta N_{S\to E}(t) \sim \text{Binomial}(S(t), p_{S\to E}(t)).$$

Exposed, latent infections (E) becoming infectious (I and A) Exposed and latent individuals will become infectious and transition to the observed infected state (I) or the unobserved infected (A) state.

The total rate of departures from E is equal to the sum of the rate of departures to I, $\frac{r_t}{d_{EI}}$, and the rate of departures to A, $\frac{1-r_t}{d_{EI}}$, which is simply $\frac{1}{d_{EI}}$. In each time step Δt, each exposed individual has a time-independent probability of becoming infectious based on this rate, $p_{E\to}(t) = 1 - e^{-\frac{1}{d_{EI}}\Delta t}$. The relative probability that a departure out of E is to I is equal to r_t, and to A is $1 - r_t$. With these probabilities we can find the number of individuals leaving E and going to I and A over time interval $[t, t + \Delta t)$, $(\Delta N_{E\to I}(t), \Delta N_{E\to A}(t))$, as

$$(\Delta N_{E\to I}(t), \Delta N_{E\to A}(t)) \sim \text{Multinomial}(E(t), (r_t)p_{E\to}(t), (1 - r_t)p_{E\to}(t)).$$

Infection (I) to Hospitalization (H) or Recovery (R) We model transitions for infectious individuals to recovery (R) or hospitalization (H).

The total rate of departures out of I is equal to the sum of the rate of departures to H, $\frac{\alpha_t}{d_{IH}}$, and the rate of departures to R, $\frac{1-\alpha_t}{d_{IR}}$. The probability that an infected individual transitions out of I in each time step is then found as $p_{I\to} = 1 - e^{-(\frac{\alpha_t}{d_{IH}}+\frac{1-\alpha_t}{d_{IR}})\Delta t}$. The relative probability that a departure is to H is equal to $\frac{\frac{\alpha_t}{d_{IH}}}{\frac{\alpha_t}{d_{IH}}+\frac{1-\alpha_t}{d_{IR}}}$, and to R is $\frac{\frac{1-\alpha_t}{d_{IR}}}{\frac{\alpha_t}{d_{IH}}+\frac{1-\alpha_t}{d_{IR}}}$. The number of infected individuals that become hospitalized in each time step, $\Delta N_{I\to H}$, and that recover, $\Delta N_{I\to R}$, are then described by the multinomial distribution,

$$(\Delta N_{I\to H}(t), \Delta N_{I\to R}(t)) \sim \text{Multinomial}(I(t), \frac{\frac{\alpha_t}{d_{IH}}}{\frac{\alpha_t}{d_{IH}}+\frac{1-\alpha_t}{d_{IR}}}p_{I\to}, \frac{\frac{1-\alpha_t}{d_{IR}}}{\frac{\alpha_t}{d_{IH}}+\frac{1-\alpha_t}{d_{IR}}}p_{I\to}).$$

Transitions out of hospital (H) and ICU (Q) We model transitions out of hospital H and out of the ICU Q in the same way as transitions out of I, each of which go to two compartments.

The probability of transition in one time step out of H is

$$p_{H\to}(t) = 1 - e^{-(\frac{\kappa_t}{d_{HQ}}+\frac{1-\kappa_t}{d_{HR}})\Delta t}.$$

The random variable for the number of hospitalized individuals that go to the ICU, $\Delta N_{H\to Q}(t)$, and that recover, $N_{H\to R}(t)$, is

$$(\Delta N_{H\to Q}(t), \Delta N_{H\to R}(t)) \sim \text{Multinomial}(H(t), \frac{\frac{\kappa_t}{d_{HQ}}}{\frac{\kappa_t}{d_{HQ}}+\frac{1-\kappa}{d_{HR}}}p_{H\to}(t), \frac{\frac{1-\kappa_t}{d_{HR}}}{\frac{\kappa_t}{d_{HQ}}+\frac{1-\kappa_t}{d_{HR}}}p_{H\to}(t)).$$

The total rate of transitions out of the ICU, to death (D) or recovery, is

$$p_{Q\to}(t) = 1 - e^{-(\frac{\delta_t}{d_{QD}}+\frac{1-\delta_t}{d_{QR}})\Delta t}$$

The random variable for the number of individuals in ICU that die, $\Delta N_{Q\to D}(t)$, and that recover, $N_{Q\to R}(t)$, is found as

$$(\Delta N_{Q\to D}(t), \Delta N_{Q\to R}(t)) \sim \text{Multinomial}(Q(t), \frac{\frac{\delta_t}{d_{QD}}}{\frac{\delta_t}{d_{QR}}+\frac{1-\delta_t}{d_{QR}}}p_{Q\to}(t), \frac{\frac{1-\delta_t}{d_{QR}}}{\frac{\delta_t}{d_{QD}}+\frac{1-\delta_t}{d_{QR}}}p_{Q\to}(t)).$$

Transitions out of unobserved infected state (A) We assume all unobserved infected cases will recover, at the rate $\frac{1}{d_{IR}}$. We model the probability of recovery in one time step as $p_{A\to}(t) = 1 - e^{-\frac{1}{d_{IR}}\Delta t}$. The number of recoveries moving from A to R in each time step is then found as

$$\Delta N_{A\to R}(t) \sim \text{Binomial}(A(t), p_{A\to}(t)).$$

1.5.2 System of equations for state variables and advancing the model

The counting process random variables are coupled to the state variables via the following identities:

$$\begin{aligned}
\Delta S(t) &= -\Delta N_{S\to E}(t)\\
\Delta E(t) &= \Delta N_{S\to E}(t) - \Delta N_{E\to I}(t) - \Delta N_{E\to A}(t)\\
\Delta I(t) &= \Delta N_{E\to I}(t) - \Delta N_{I\to H}(t) - \Delta N_{I\to R}(t)\\
\Delta A(t) &= \Delta N_{E\to A}(t) - \Delta N_{A\to R}(t)\\
\Delta H(t) &= \Delta N_{I\to H}(t) - \Delta N_{H\to Q}(t) - \Delta N_{H\to R}(t)\\
\Delta Q(t) &= \Delta N_{H\to Q}(t) - \Delta N_{Q\to D}(t) - \Delta N_{Q\to R}(t)\\
\Delta D(t) &= \Delta N_{Q\to D}(t)\\
\Delta R(t) &= \Delta N_{I\to R}(t) + \Delta N_{H\to R}(t) + \Delta N_{Q\to R}(t).
\end{aligned}$$

To simulate from this system we employ an Euler numerical scheme for Markov process compartment models with stochastic rates [6]. We sample the transitions from the counting processes $\Delta N_{X_i\to X_j}(t)$, and update each state variable, representing the number of individuals in each compartment at time t, to reflect these counts.

1.5.3 Cumulative and daily counts

The dynamics of the stochastic model are advanced by updating state variables representing the "current census" of individuals in each compartment X_i. Observed COVID-19 infection data, however, is available as daily or cumulative counts. We therefore also keep track of auxiliary count variables representing the daily new counts or cumulative infection counts (observed data described in Section 2.2).

For cumulative counts we have, based on the counting processes for transitions between compartments already defined:

$$I_{cum}(t+1) = I_{cum}(t) + \Delta N_{E\to I}(t)$$

where the I_{cum} represents the cumulative version of each state variable I. Daily count I_{new} can be worked out from the cumulative counts as $I_{new}(t+1) = I_{cum}(t+1) - I_{cum}(t)$.

1.6 Solving for the basic reproduction number, R_0

The basic reproductive number, $R0$, is a dimensionless parameter defined as the average number of secondary infections produced by a single infected individual during that individual's entire period of infection, in a completely susceptible population and no interventions to reduce the spread [9]. While not a parameter of the system of state variables, $R0$ is a parameter of interest because it provides a measure of the likelihood that an epidemic will occur within a population.

In a deterministic model, $R0$ represents an epidemic threshold for which values of $R0 < 1$ indicate a lack of disease spread, and values of $R0 > 1$ are consistent with epidemic spread. For a stochastic model, if $R0 > 1$ the probability that an epidemic will develop is less than 1, and if $R0 < 1$, extinction of the infection will occur with a probability less than 1. On average, however, no epidemic will occur if $R0 \leq 1$ [10]. Therefore, estimates of $R0$ provide clearly communicable insight regarding the degree of intensity of interventions required to achieve control [9].

For a simple SIR model, $R0$ is a simple expression equal to the product of the number of infections per time for an infected individual, i.e. the transmission rate β, and the average duration of the infectious period, e.g. d_I, that is,

$$R0 = \frac{\text{infections}}{\text{time}} \times \frac{\text{time}}{\text{infection}} = \beta d_I.$$

Because our model involves multiple infectious classes with different rates of infection duration, the average number of individuals an infected individual infects is a more complex expression. We use the *Next Generation Matrix* (NGM) [11] approach to find the expression for $R0$. The NGM approach involves computing $R0$ by linearizing the ODE model in Section 1.4 around the infection-free steady state and identifying conditions that guarantee increases in infected states [11]. We focus on the *infection subsystem*, i.e. the subset of ODEs that describe the production of new infections and changes in the states of already existing infections only, which is

$$\begin{aligned}
dE/dt &= \beta_0 S(I+A) - \tfrac{1}{d_{EI}}E\\
dA/dt &= \tfrac{1-r_0}{d_{EI}}E - \tfrac{1}{d_{IR}}A\\
dI/dt &= \tfrac{r_0}{d_{EI}}E - (\tfrac{\alpha_0}{d_{IH}} + \tfrac{1-\alpha_0}{d_{IR}})I,
\end{aligned}$$

where β_0, r_0, and α_0 represent the initial estimated values of these parameters.

Setting $X = (\frac{dE}{dt}, \frac{dA}{dt}, \frac{dI}{dt})^\intercal$, we write the linearized infection subsystem in the form

$$\frac{dX}{dt} = (T+\Sigma)X,$$

where T corresponds to all epidemiological events leading to new infections, and Σ corresponds to all epidemiological events relating to transitions in or out of these infected states. We write the new infection matrix T as

$$T = \begin{pmatrix} 0 & \beta_0 & \beta_0 \\ 0 & 0 & 0 \\ 0 & 0 & 0 \end{pmatrix}$$

and the new transmission matrix Σ as

$$\Sigma = \begin{pmatrix} -\frac{1}{d_{EI}} & 0 & 0 \\ \frac{r_0}{d_{EI}} & -(\frac{\alpha_0}{d_{IH}} + \frac{1-\alpha_0}{d_{IR}}) & 0 \\ \frac{1-r_0}{d_{EI}} & 0 & -\frac{1}{d_{IR}} \end{pmatrix}.$$

Diekmann et al. [11] show that $R0$ can be found as the dominant eigenvalue of the next generation matrix $K_L = -T\Sigma^{-1}$, or equivalently, the dominant eigenvalue of $K = E^\intercal\Sigma^{-1}E$, where E is an auxiliary matrix whose columns consist of unit vectors relating to the non-zero rows of T. E, which in this case is equal to $E^\intercal = \begin{bmatrix} 1 & 0 & 0 \end{bmatrix}$, is included to reduce dimensionality.

We find Σ^{-1} as

$$\Sigma^{-1} = \begin{pmatrix} d_{EI} & 0 & 0 \\ \frac{-r_0}{\frac{\alpha_0}{d_{IH}} + \frac{1-\alpha_0}{d_{IR}}} & \frac{1}{\frac{\alpha_0}{d_{IH}} + \frac{1-\alpha_0}{d_{IR}}} & 0 \\ -(1-r_0)d_{IR} & 0 & d_{IR} \end{pmatrix}.$$

We have $E^\intercal T = \begin{bmatrix} 0 & \beta_0 & \beta_0 \end{bmatrix}$, and $\Sigma^{-1}E = \begin{bmatrix} d_{EI} & \frac{-r_0}{\frac{\alpha_0}{d_{IH}} + \frac{1-\alpha_0}{d_{IR}}} & -(1-r_0)d_{IR} \end{bmatrix}^\intercal$. We can then find the next generation matrix as,

$$K = -E^\intercal T\Sigma^{-1}E = \beta_0 \left[\frac{-r_0}{\frac{\alpha_0}{d_{IH}} + \frac{1-\alpha_0}{d_{IR}}} + (1-r_0)d_{IR} \right].$$

$R0$ is the dominant eigenvalue of K, which is simply equal to K in this case. Therefore,

$$R0 = \beta_0 \left[\frac{r_0}{\frac{\alpha_0}{d_{IH}} + \frac{1-\alpha_0}{d_{IR}}} + (1-r_0)d_{IR} \right].$$

We note that the NGM approach for deriving $R0$ is based on the deterministic ODE equations, while we use this parameter with the stochastic model. We justify deriving it from the deterministic model because for large population sizes N, the mean of the stochastic equations will converge on the deterministic model equations [10, 12].

Given that $R0$ is the value of the reproductive number at the beginning of the pandemic period before modifications, we define the time-varying reproductive number as R_t.

Additionally, the reproduction number will decrease as natural immunity increases, reflecting the fact that as the proportion of susceptible individuals decreases ($S(t)/P$), disease transmission slows [13]. We therefore define the time-varying effective reproductive number, accounting for both the community transmission reduction factor and the decreasing proportion of susceptible individuals, as $R_{eff,t} = R_t \frac{S(t)}{N}$.

2 Parameter Estimation

Transmission rate parameters we model as fixed values taken from the literature (Table 2). The remaining five model parameters, $\{\beta_t, r_t, \alpha_t, \kappa_t, \delta_t\}$ (Table 3), must be estimated with data. To this set of five parameters we add an additional parameter, t_0, representing the starting time of the epidemic in LAC; that is, the very first case of COVID-19, which may have been unobserved. This parameter links the epidemic model to the observed count data based on calendar dates. In addition, because

prior information from existing studies on the reproductive number R_t is more readily available than for β_t, we estimate R_t from data and then use equation 1.6 to obtain β_t from R_t. Therefore the estimated parameter set is $\theta = \{R_t, r_t, \alpha_t, \kappa_t, \delta_t, t_0\}$.

We use a two-step process to define unimodal posterior distributions and achieve model convergence, first performing a broad grid search to define a narrow range for each parameter, and second using Approximate Bayesian Computation (ABC) techniques with prior distributions set by the ranges defined by the grid search to estimate the final posterior distribution for each parameter. External data sources were used to specify the limits of the parameter space for the grid search. In the following we describe the data sources used to inform the grid search space for each parameter, and the resulting prior parameter distributions chosen to use in ABC.

2.1 Specifying prior parameter distributions

2.1.1 Basic Reproduction Number, R_t

We base the prior distribution for $R0$ on values for $R0$ estimated from other published studies on COVID-19. A review of $R0$ values estimated for COVID-19 across studies involving different locations, time periods, and modeling approaches reported values in the range of [1.40-6.49], with a mean of 3.28 [14]. Values for $R0$ in SEIR models are also higher than those in *SIR* models, as has been demonstrated by previous researchers [15]; for example, Bertozzi et al. [16] demonstrate an estimated $R0$ for California of 2.4 using a SIR model and 4.9 using an SEIR model. After performing a broad grid search across a parameter space from $[3,5]$, we model a prior distribution for $R0$ in ABC as a normal distribution centered around 3.5, with a standard deviation chosen such that 95% of the area of the distribution is within approximately ± 0.25 of the mean. The modeled prior distribution for $R0$ is therefore:

$$R0 \sim N(3.5, 0.2)$$

2.1.2 Time-varying Reproduction Number after Interventions, R_t

Geolocation trace data from smartphones, i.e. mobility data, can be used estimate changes in distances travelled, commuting, time spent outside of home, visits to specific types of venues, and encounter rates in the community. These data sources, which represent contact rates in the population, can be used as a proxy to estimate changes in contact rate and equivalently in β_t and R_t over time [17].

We use mobility data to inform the magnitude and the timing of inflection points in the factor reduction in R_t. We reference mobility data for LAC from a dashboard provided by Unacast, a company that builds and maintains anonymized mobility datasets based on geolocation data aggregated from millions of users across the U.S. [18]. Unacast provides three metrics: the *Change in Average Mobility (Based on Distance Traveled)*, the *Change in Non-Essential Visits*, and the *Difference in Encounter Density*. We reference the metrics *Change in Average Mobility (Based on Distance Traveled)* and *Difference in Encounter Density* (Figure 1) because these both inform the changes and timing of changes in contact rates, in different ways [17].

Across these two metrics we observe the following trends in $\mu(t)$: (i) a sharp initial descent from the original contact rate beginning around March 12 2020 (a few days before county-wide quarantine policy was announced on March 15), and leveling out around March 28; (ii) a steady plateau during the initial period of the lockdown from around March 28 - April 26; (iii) a steady incline in contact rates from April 27 - May 15 (May 8th being the end of the lockdown); (iv) a lower plateau beginning August 15 (when schools opened), and (v) an increase beginning October 15.

As noted in the main text, an increase beginning in October and throughout the third wave of the epidemic in LAC is not visible on the mobility metrics, but is required for the model to achieve accurate fits to data; this likely reflects the fact that the infection rate is dependent on both the contact rate and the probability of transmission given contact and may include decreasing NPI adherence, more time spend indoors, and other conditions that may increase transmissiblility of the virus. Thus, for dates after October 15, 2020, we stopped using mobility data to inform the grid search space for R_t and instead set this equal to $1 < R_t < R0$, for dates corresponding to increasing infection trends.

Based on the results from the grid search step, we develop a distribution with the change points in time given in Table 4. A piecewise linear function is interpolated between dates.

2.1.3 Fraction of observed infections r_t

Grid search was not performed for r_t; this was only estimated within the ABC framework. The prior distribution for r_t is estimated on April 15 (r_1), and August 15, 2020 (r_2). We interpolate a linear change between the values on the two dates, and assume that r_t before April 15 is equal to r_1 and after August 15 is equal to r_2, i.e. the distribution shown in Table 5. The prior for r_1 is allowed to vary over an interval truncated by minimum and maximum values calculated by seroprevalence studies performed by the CDC in March - early May, which were found to be as few as 34.7 times as many infections as observed cases, and as many as 3.2 times as many cases observed as estimated, in the study regions [19]. We allow the second date r_t to vary over a much wider interval since we do not have prior information corresponding to this time period.

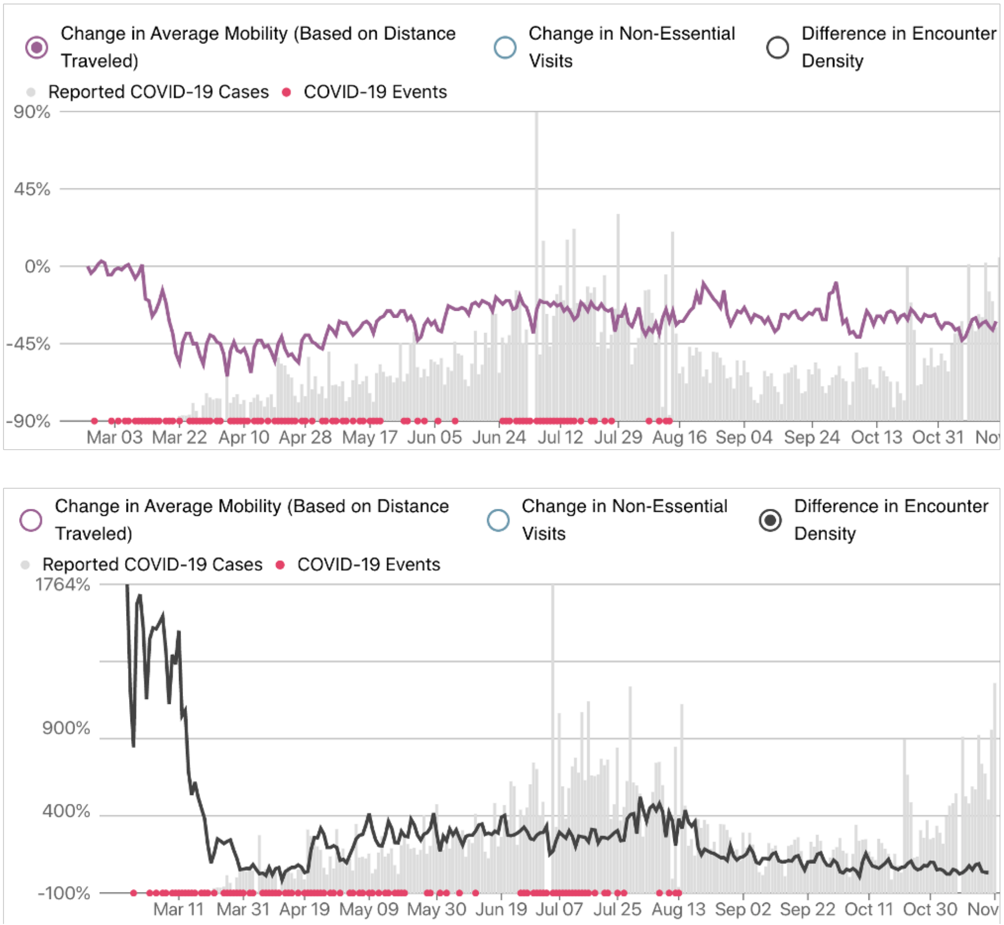

Figure 1. The *Change in Average Mobility (Based on Distance Traveled)* and the *Difference in Encounter Density* metric for Los Angeles County from March 1 - Nov 15, 2020 from the the Unacast dashboard [18].

date	prior r_t
2020-03-01	$\sim N(3.65, 0.2)$
2020-03-12	$\sim N(3.65, 0.2)$
2020-03-27	$\sim N(0.77, 0.11)$
2020-04-27	$\sim N(0.77, 0.11)$
2020-05-15	$\sim N(1.2, 0.12)$
2020-07-01	$\sim N(1.2, 0.12)$
2020-08-15	$\sim N(0.77, 0.11)$
2020-10-13	$\sim N(0.77, 0.11)$
2020-10-14	$\sim N(2.01, 0.13)$
2021-01-01	$\sim N(2.01, 0.13)$
2021-01-02	$\sim N(2.51, 0.14)$
2021-01-03	$\sim N(2.51, 0.14)$
2021-01-20	$\sim N(1.3, 0.12)$
2021-01-16	$\sim N(1.3, 0.12)$
2021-03-01	$\sim N(1.3, 0.12)$

Table 4. Prior distribution for the time-varying reproductive number r_t.

date	r_t
2020-03-01	$\sim U(0.20, 0.1)$
2020-04-15	$\sim U(0.20, 0.1)$
2020-08-15	$\sim U(0.50, 0.1)$
2021-03-01	$\sim U(0.50, 0.1)$

Table 5. Prior distribution for the fraction of observed cases over all infections, r_t.

date	prior α_t	prior κ_t	prior δ_t
2020-03-01	$\sim N(0.375, 0.01)$	$\sim N(0.35, 0.01)$	$\sim N(0.6, 0.01)$
2020-03-01	$\sim N(0.375, 0.01)$	$\sim N(0.35, 0.01)$	$\sim N(0.6, 0.01)$
2020-04-30	$\sim N(0.375, 0.01)$	$\sim N(0.35, 0.01)$	$\sim N(0.6, 0.01)$
2020-05-01	$\sim N(0.375, 0.01)$	$\sim N(0.35, 0.01)$	$\sim N(0.6, 0.01)$
2020-05-15	$\sim N(0.15, 0.01)$	$\sim N(0.2, 0.01)$	$\sim N(0.55, 0.01)$
2020-06-15	$\sim N(0.15, 0.01)$	$\sim N(0.2, 0.01)$	$\sim N(0.55, 0.01)$
2020-07-01	$\sim N(0.18, 0.01)$	$\sim N(0.25, 0.01)$	$\sim N(0.605, 0.01)$
2020-07-15	$\sim N(0.18, 0.01)$	$\sim N(0.25, 0.01)$	$\sim N(0.605, 0.01)$
2020-08-01	$\sim N(0.15, 0.01)$	$\sim N(0.2, 0.01)$	$\sim N(0.55, 0.01)$
2020-08-20	$\sim N(0.15, 0.01)$	$\sim N(0.2, 0.01)$	$\sim N(0.55, 0.01)$
2020-10-01	$\sim N(0.15, 0.01)$	$\sim N(0.2, 0.01)$	$\sim N(0.55, 0.01)$
2020-10-06	$\sim N(0.0975, 0.01)$	$\sim N(0.16, 0.01)$	$\sim N(0.65, 0.01)$
2021-01-01	$\sim N(0.0975, 0.01)$	$\sim N(0.16, 0.01)$	$\sim N(0.65, 0.01)$
2021-01-05	$\sim N(0.0975, 0.01)$	$\sim N(0.16, 0.01)$	$\sim N(0.65, 0.01)$
2021-03-17	$\sim N(0.0975, 0.01)$	$\sim N(0.16, 0.01)$	$\sim N(0.65, 0.01)$
2021-12-15	$\sim N(0.0975, 0.01)$	$\sim N(0.16, 0.01)$	$\sim N(0.65, 0.01)$

Table 6. Prior distributions for the time-varying probabilities of each disease stage over time: probability of hospitalization given infection, α_t; probability of ICU admission given hospitalization, κ_t; probability of death given ICU admission, δ_t.

2.1.4 Probabilities of disease stage progression

Grid search ranges for the parameters representing the probabilities of disease stage progression, α_t, κ_t, and δ_t, were informed by the ratios of the observed numbers of infections, hospitalizations, and deaths in LAC. Specifically, key change points in these ratios informed our modeling of change points in the distribution of the three parameters over time. At each change point (the date column in table 6) we re-estimated the three parameters by first performing the broad grid search step over a wide range for each parameter ([.05,.9]). After choosing a mode returned by the grid search posterior distribution for each parameter at each time point, we set the prior parameter distributions as specified in table 6.

2.1.5 Starting time, t_0

The first observed case of COVID-19 in LAC is registered as occurring on January 25 2020, and the epidemic began to grow exponentially in early March 2020 [20]. It is likely that the epidemic in LAC had multiple starting points. We therefore model t_0 as a uniform distribution with the unobserved first initial case beginning anywhere between January 1 and February 15 2020.

2.2 Variable observations used for parameter estimation

The model was fit to the daily and cumulative count of observed infections and deaths, and current numbers in-hospital and in-ICU, coming from the GitHub page of the Los Angeles Times (LA Times) Data and Graphics Department [21]. The infection and death data is sourced from reports logged by LA Times reporters and editors based on reports from the LAC Department of Public Health. The in-hospital and in-ICU data was sourced by the LA Times directly from the California Department of Public Health's Open Data Portal [22]. We use the total of both confirmed and suspected COVID-19 patients in hospital or ICU.

2.2.1 Count variables used in model estimation

We combine the new and cumulative counts of infections and deaths and current numbers in-hospital and in-ICU into a single computed summary statistic to use in ABC, $Summ(\Phi) = \{I_{new}(t), I_{cum}(t), D_{new}(t), D_{cum}(t), H(t), Q(t)\}$, where Φ represents the observed data. We truncate the data such that we do not include infection observations from early in the epidemic, when observations are more likely to be incomplete and unreliable. Specifically, we compute the summary statistic, $Summ(\Phi)$, for

the data Φ: $Summ(\Phi) = \{I_{cum}(t > \text{March 15}), H(t > \text{March 20}), Q(t > \text{March 20}), D_{cum}(t > \text{March 25})\}$.

2.3 Running the model

2.3.1 Uncertainty quantification

We use ABC to estimate all model parameters simultaneously, producing joint posterior probability estimates over all parameters. In forward simulations of our model, we simulate trajectories with parameter values coming from this joint posterior distribution, rather than a single value of a parameter. Uncertainty is also contributed by the stochastic differential equations, which model stochasticity in numbers of transitions between compartments.

To produce uncertainty estimates, we estimate credible intervals (CIs) for all model variables and estimated parameters by quantifying uncertainty from two sources: variability due to joint estimated parameter values, and variability due to the stochastic variability between model runs with the same parameters. We aggregate simulations from 100 jointly estimated parameter sets. For each parameter set, we simulate 20 realizations of the stochastic epidemic model, resulting in 2000 total realizations. We pool together all simulations and report their median and 2.5th/97.5th percentiles.

2.3.2 Model implementation

The model was implemented in R (version 3.6.3).

Epidemic model initial values In our experiments, model time $t = 0$ is set to the ABC-estimated time for t_0, which is linked to the calendar date of around January 15, 2020, though this will vary across the estimated parameter sets. We set $S(0)$ is equal to 10 million, approximately the population of LAC; $I(0) = 1$, $E(0) = 10$; and all other compartments initially empty.

Parameter estimation implementation details We apply the R package EasyABC [23] and the algorithm *Marjoram*, which implements the algorithm of Marjoram et al. (2003) [24] with improvement steps by Wegmann et al. (2009) [25]. The arguments that need to be specified are the prior parameter distributions, the original data Φ, the epidemic model to simulate data Φ^* from, and the function that computes summary statistics $Summ(\Phi)$. The additional parameters that need to be specified are the number n of simulations to perform in the calibration step; the number of parameter values to accept N, the *tolerance quantile* η that helps to determine the tolerance level ξ; and the *scale factor* ρ that helps to determine the size of the proposal range.

We set $n = 100,000$ burn-in simulations, $N = 1000$ sets of jointly estimated parameter values to save, and use the default values for tolerance quantile of $\eta = 0.01$ and scale factor of $\rho = 1$. We test the model for convergence by running the parameter estimation procedure multiple times with different seeds and verifying similarity across variable outputs. Prior parameter distributions were adjusted such that convergence is achieved.

2.4 Calculating population-wide CFR_t and IFR_t

For each simulated model realization, we find the CFR_t and IFR_t as the estimated number of deaths (D) for each profile over estimated observed infections (I) for each profile, and number of deaths over total infections ($I + A$), respectively. Repeating across the 2000 model realizations achieves the 95% CI.

3 Epidemic model results

3.1 Estimated timeseries of numbers in infection states

This section provides close-ups of the model-estimated timeseries of numbers in infection states relative to key dates and COVID-19 policy decisions in LAC. Available data is shown for new infections, new deaths, and current numbers in-hospital and in-ICU. Model-estimated current vs. total infections, although without data, are also shown. For each figure the the model-estimated median curves are plotted along with the 50th% (dark shading) and 95% CI (light shading).

3.2 Parameter estimates

Estimates of each of the five model parameters, and the population-wide CFR_t and IFR_t, at two week intervals between March 1, 2020 and March 1, 2021 are shown in Table 7.

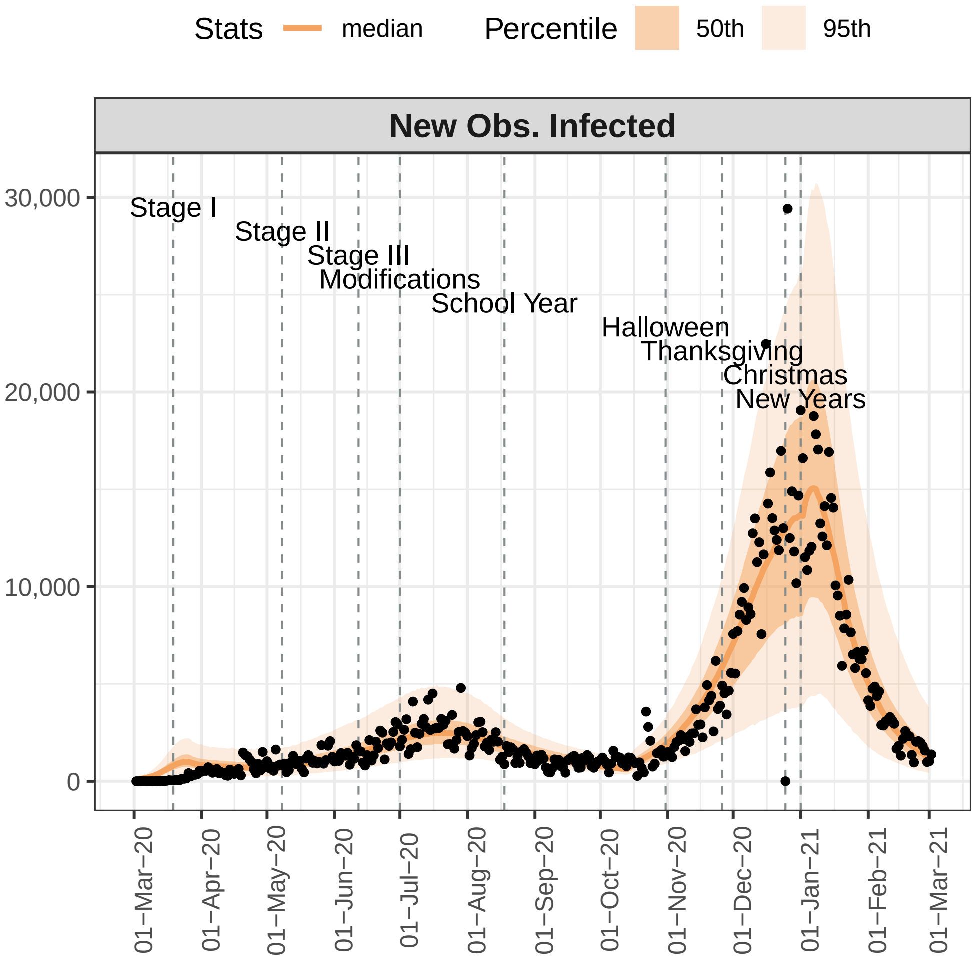

Figure 2. New infections, I_{new}.

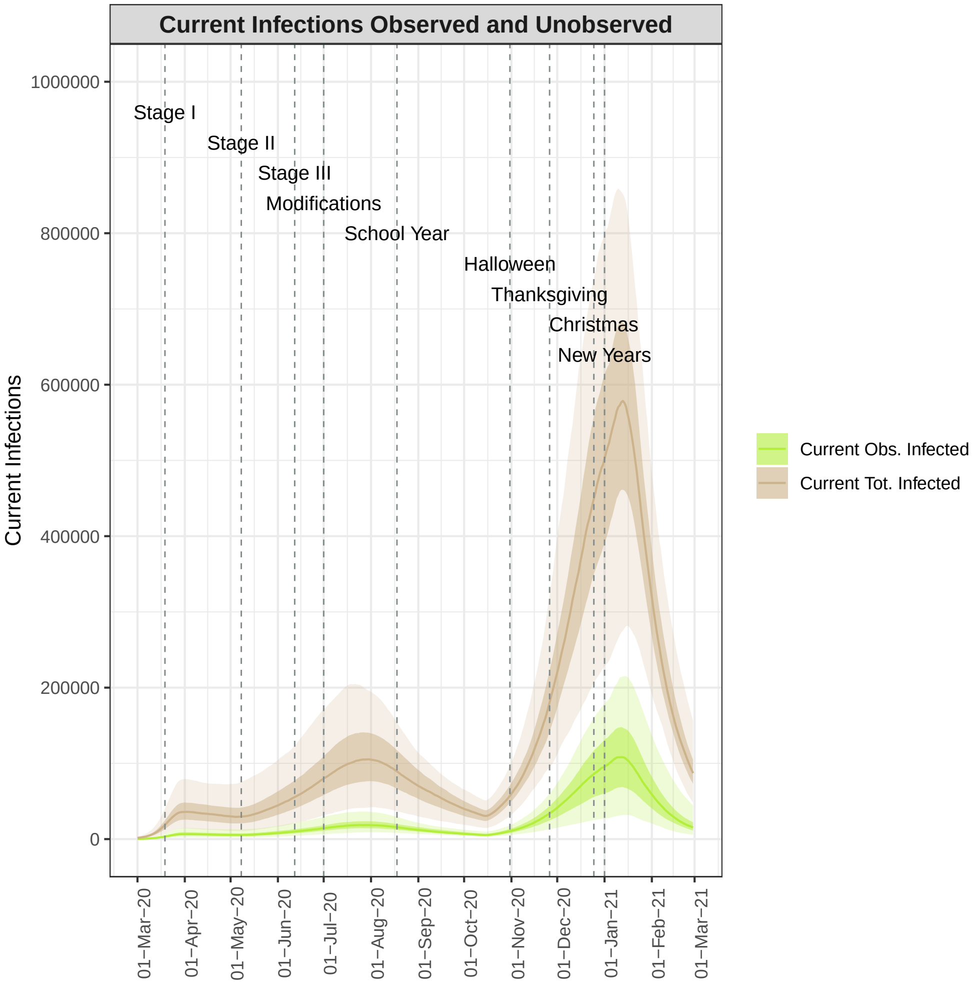

Figure 3. Current observed infections, I, and total infections, $I+A$.

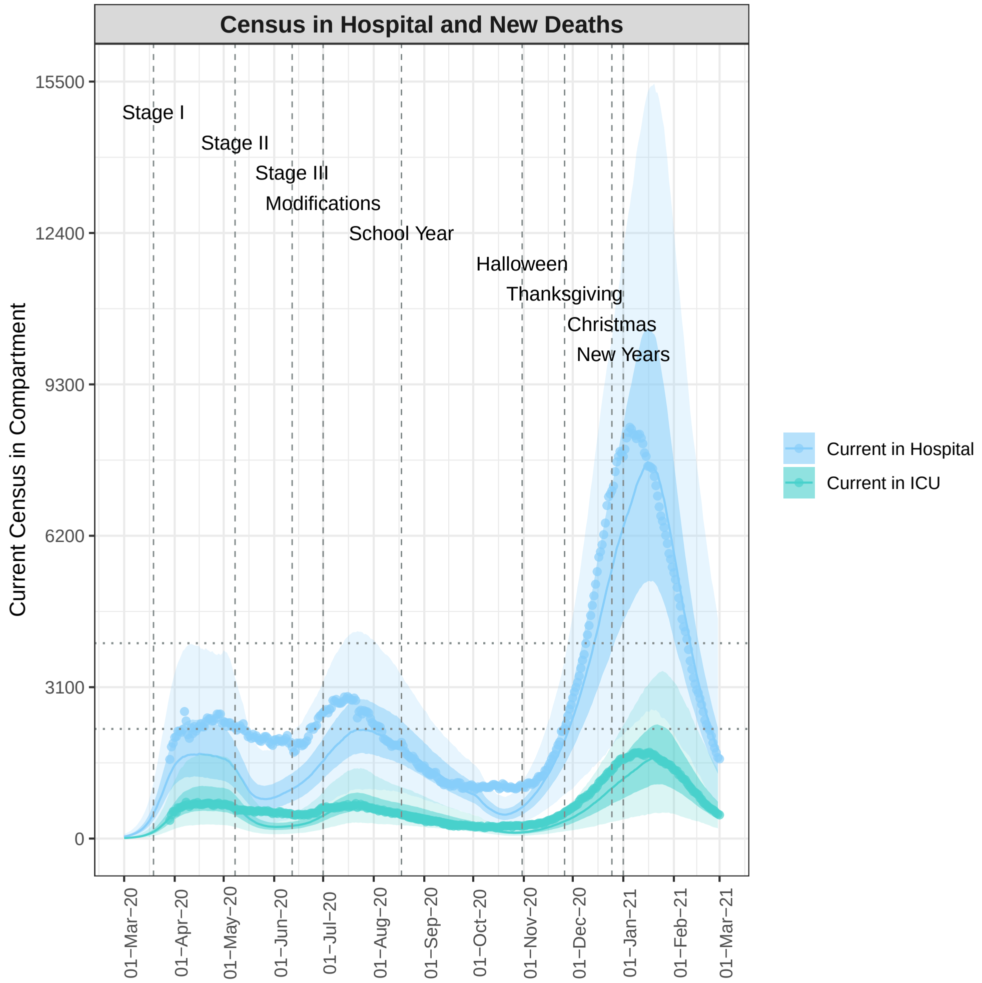

Figure 4. Current in-hospital, H, and in-ICU, Q. The first of the two horizontal dotted lines shows the approximate hospital capacity limit, while the second shows the ICU capacity limit.

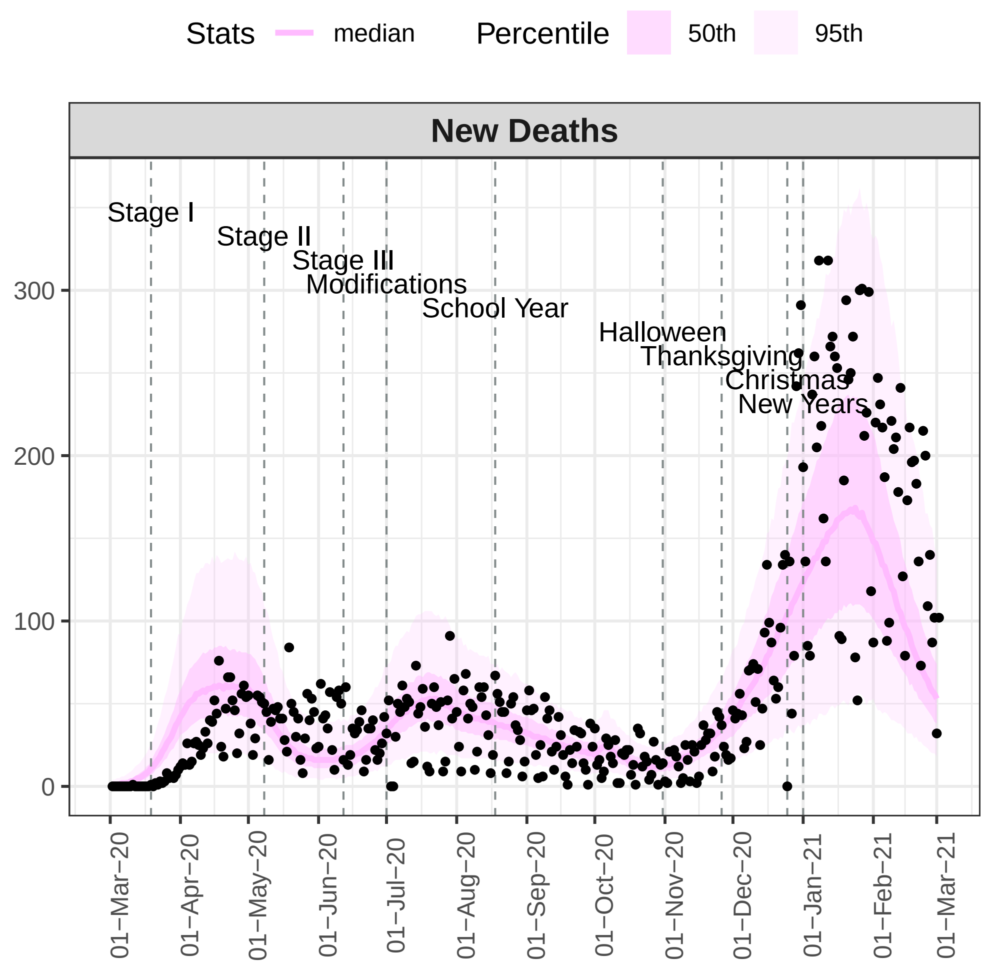

Figure 5. New deaths, D.

date	R_t	R_t Effective	r_t	α_t	κ_t	δ_t	CFR_t %	IFR_t %
**3/1/20**	3.69 (3.6,3.82)	3.69 (3.6,3.82)	0.19 (0.12,0.26)	0.349 (0.322,0.38)	0.327 (0.309,0.348)	0.506 (0.452,0.565)	1.21 (0.21,2.42)	0.24 (0.03,0.54)
**3/15/20**	3.13 (3.04,3.24)	3.12 (3.02,3.24)	0.19 (0.12,0.26)	0.349 (0.322,0.38)	0.327 (0.309,0.348)	0.506 (0.452,0.565)	1.21 (0.77,1.62)	0.24 (0.08,0.43)
**4/1/20**	0.88 (0.77,0.95)	0.87 (0.76,0.95)	0.19 (0.12,0.26)	0.349 (0.322,0.38)	0.327 (0.309,0.348)	0.506 (0.452,0.565)	2.31 (1.77,2.67)	0.46 (0.17,0.75)
**4/15/20**	0.88 (0.77,0.95)	0.87 (0.75,0.95)	0.19 (0.12,0.26)	0.349 (0.322,0.38)	0.327 (0.309,0.348)	0.506 (0.452,0.565)	4.18 (3.27,4.74)	0.83 (0.31,1.36)
**5/1/20**	0.97 (0.84,1.05)	0.95 (0.8,1.04)	0.23 (0.15,0.31)	0.349 (0.322,0.38)	0.327 (0.309,0.348)	0.506 (0.452,0.565)	5.56 (4.35,6.3)	1.1 (0.41,1.81)
**5/15/20**	1.26 (1.06,1.39)	1.23 (1.01,1.37)	0.27 (0.18,0.36)	0.153 (0.118,0.19)	0.196 (0.143,0.234)	0.526 (0.503,0.543)	5.86 (4.61,6.62)	1.16 (0.43,1.91)
**6/1/20**	1.26 (1.06,1.39)	1.21 (0.99,1.37)	0.31 (0.21,0.41)	0.153 (0.118,0.19)	0.196 (0.143,0.234)	0.526 (0.503,0.543)	4.97 (3.86,5.64)	0.99 (0.36,1.64)
**6/15/20**	1.26 (1.06,1.39)	1.2 (0.97,1.36)	0.34 (0.23,0.45)	0.153 (0.118,0.19)	0.196 (0.143,0.234)	0.526 (0.503,0.543)	4.18 (3.2,4.81)	0.84 (0.3,1.4)
**7/1/20**	1.26 (1.06,1.39)	1.18 (0.94,1.36)	0.39 (0.26,0.5)	0.184 (0.142,0.228)	0.246 (0.179,0.293)	0.579 (0.553,0.597)	3.51 (2.69,4.18)	0.71 (0.26,1.19)
**7/15/20**	1.14 (0.97,1.25)	1.05 (0.83,1.22)	0.42 (0.28,0.54)	0.184 (0.142,0.228)	0.246 (0.179,0.293)	0.579 (0.553,0.597)	3.2 (2.46,3.89)	0.64 (0.24,1.09)
**8/1/20**	1 (0.86,1.09)	0.9 (0.7,1.05)	0.46 (0.31,0.6)	0.153 (0.118,0.19)	0.196 (0.143,0.234)	0.526 (0.503,0.543)	3 (2.3,3.68)	0.6 (0.23,1.03)
**8/15/20**	0.88 (0.77,0.95)	0.78 (0.61,0.91)	0.5 (0.34,0.64)	0.153 (0.118,0.19)	0.196 (0.143,0.234)	0.526 (0.503,0.543)	2.86 (2.19,3.54)	0.58 (0.22,0.99)
**9/1/20**	0.88 (0.77,0.95)	0.77 (0.6,0.91)	0.5 (0.34,0.64)	0.153 (0.118,0.19)	0.196 (0.143,0.234)	0.526 (0.503,0.543)	2.79 (2.13,3.45)	0.56 (0.22,0.97)
**9/15/20**	0.88 (0.77,0.95)	0.76 (0.58,0.91)	0.5 (0.34,0.64)	0.153 (0.118,0.19)	0.196 (0.143,0.234)	0.526 (0.503,0.543)	2.76 (2.1,3.42)	0.55 (0.22,0.97)
**10/1/20**	0.88 (0.77,0.95)	0.76 (0.58,0.9)	0.5 (0.34,0.64)	0.153 (0.118,0.19)	0.196 (0.143,0.234)	0.526 (0.503,0.543)	2.73 (2.08,3.38)	0.55 (0.22,0.96)
**10/15/20**	2.03 (1.87,2.22)	1.73 (1.37,2.1)	0.5 (0.34,0.64)	0.1 (0.077,0.124)	0.157 (0.114,0.187)	0.737 (0.704,0.76)	2.74 (2.08,3.39)	0.55 (0.22,0.96)
**11/1/20**	2.03 (1.87,2.22)	1.69 (1.34,2.08)	0.5 (0.34,0.64)	0.1 (0.077,0.124)	0.157 (0.114,0.187)	0.737 (0.704,0.76)	2.6 (1.96,3.24)	0.52 (0.21,0.92)
**11/15/20**	2.03 (1.87,2.22)	1.63 (1.29,2.03)	0.5 (0.34,0.64)	0.1 (0.077,0.124)	0.157 (0.114,0.187)	0.737 (0.704,0.76)	2.36 (1.73,3.02)	0.47 (0.19,0.85)
**12/1/20**	2.03 (1.87,2.22)	1.51 (1.18,1.91)	0.5 (0.34,0.64)	0.1 (0.077,0.124)	0.157 (0.114,0.187)	0.737 (0.704,0.76)	2.01 (1.34,2.73)	0.4 (0.16,0.74)
**12/15/20**	2.03 (1.87,2.22)	1.33 (1.01,1.74)	0.5 (0.34,0.64)	0.1 (0.077,0.124)	0.157 (0.114,0.187)	0.737 (0.704,0.76)	1.72 (1.08,2.44)	0.34 (0.14,0.64)
**1/1/21**	2.03 (1.87,2.22)	1.05 (0.72,1.42)	0.5 (0.34,0.64)	0.1 (0.077,0.124)	0.157 (0.114,0.187)	0.737 (0.704,0.76)	1.51 (0.97,2.2)	0.29 (0.13,0.54)
**1/15/21**	1.4 (1.29,1.53)	0.57 (0.35,0.8)	0.5 (0.34,0.64)	0.1 (0.077,0.124)	0.157 (0.114,0.187)	0.737 (0.704,0.76)	1.42 (0.98,2.01)	0.28 (0.13,0.49)
**2/1/21**	1.32 (1.21,1.44)	0.45 (0.27,0.67)	0.5 (0.34,0.64)	0.1 (0.077,0.124)	0.157 (0.114,0.187)	0.737 (0.704,0.76)	1.5 (1.12,1.98)	0.29 (0.14,0.5)
**2/15/21**	1.32 (1.21,1.44)	0.42 (0.25,0.63)	0.5 (0.34,0.64)	0.1 (0.077,0.124)	0.157 (0.114,0.187)	0.737 (0.704,0.76)	1.59 (1.23,2.02)	0.31 (0.15,0.53)
**3/1/21**	1.32 (1.21,1.44)	0.41 (0.25,0.62)	0.5 (0.34,0.64)	0.1 (0.077,0.124)	0.157 (0.114,0.187)	0.737 (0.704,0.76)	1.65 (1.29,2.06)	0.32 (0.16,0.55)

Table 7. Median and 95% confidence interval (CI) of estimated model parameters and quantities over time. CFR_t and IFR_t are shown as percentages.

3.3 Posterior parameter densities

This section visualizes the density plots for the prior distribution specified for, and the estimated posterior distribution returned by, ABC parameter estimation. A single value is shown for each parameter, corresponding to the value it takes on over a specific time interval. For each parameter, the bottom subfigure shows the posterior density over its full range, and the top subfigure shows the prior density over that same range. Therefore, if the prior density has a wider distribution or a different range than the posterior, the prior density will appear truncated in the figure. The prior distributions shown were informed by the results of the broad grid search that scanned across a much wider range of each parameter space (these ranges are specified in Section 2.1).

Convergence is not reached for the broad grid search step, with multi-modal distributions returned for each parameter (not shown). By specifying a narrow prior distribution around a mode chosen from the broad grid search distribution, convergence is reached and a dominant single mode identified in the final posterior density returned by the ABC step.

Although the prior distributions used in the ABC step are narrow (with 95% of a prior parameter's value lying within $\pm 25\%$ of the mean of the chosen mode from broad grid search), they are not too narrow to allow the posterior distributions to take a different shape. All posterior distributions differ slightly from the priors; the mean of each posterior is not exactly aligned with the mean of the prior, and the standard deviations becomes narrower.

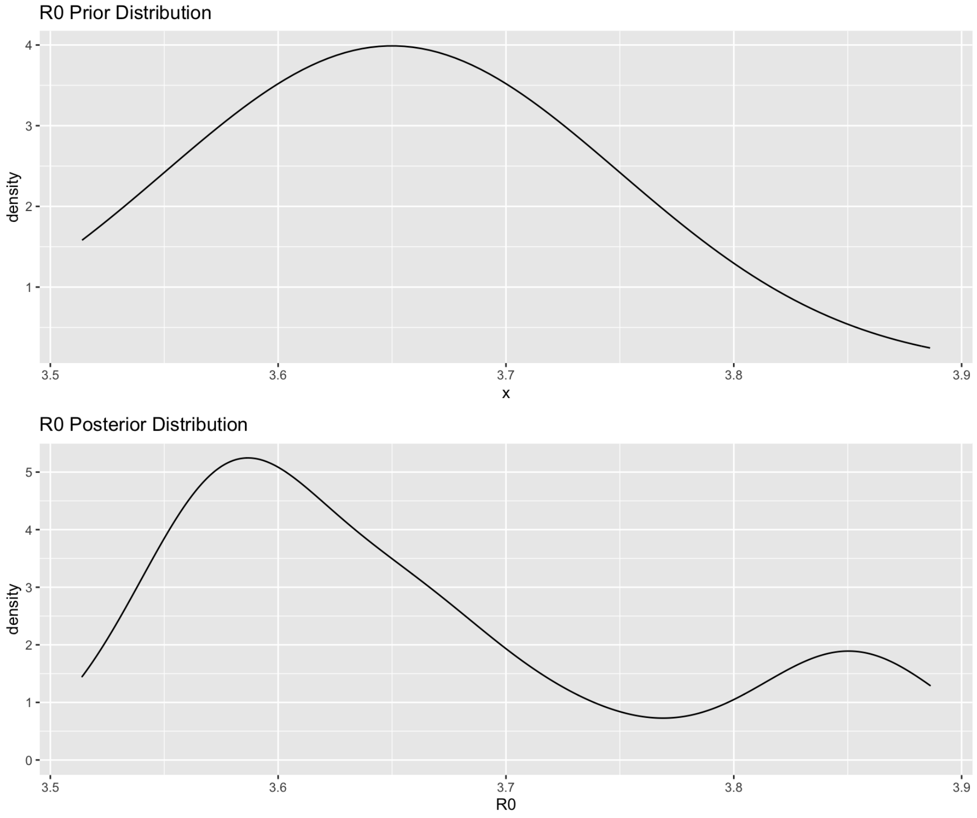

Figure 6. Basic reproductive number $R0$: value at beginning of the epidemic before interventions.

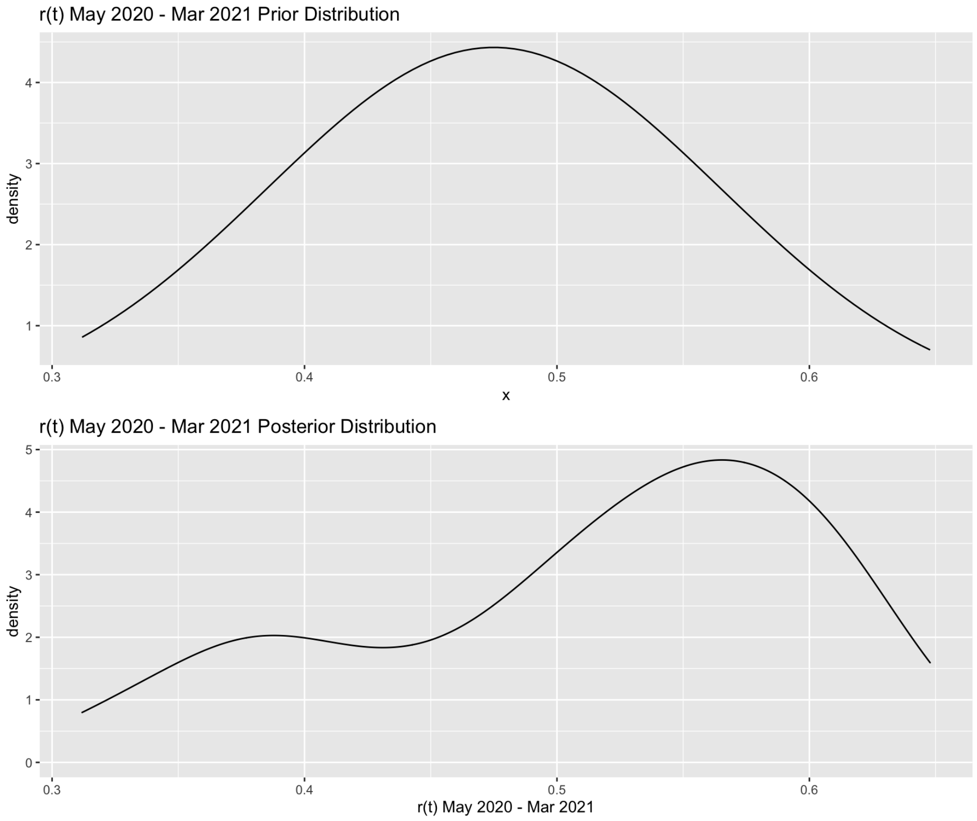

Figure 7. Fraction of infections that are observed, r_t: value during May 2020 – March, 2021.

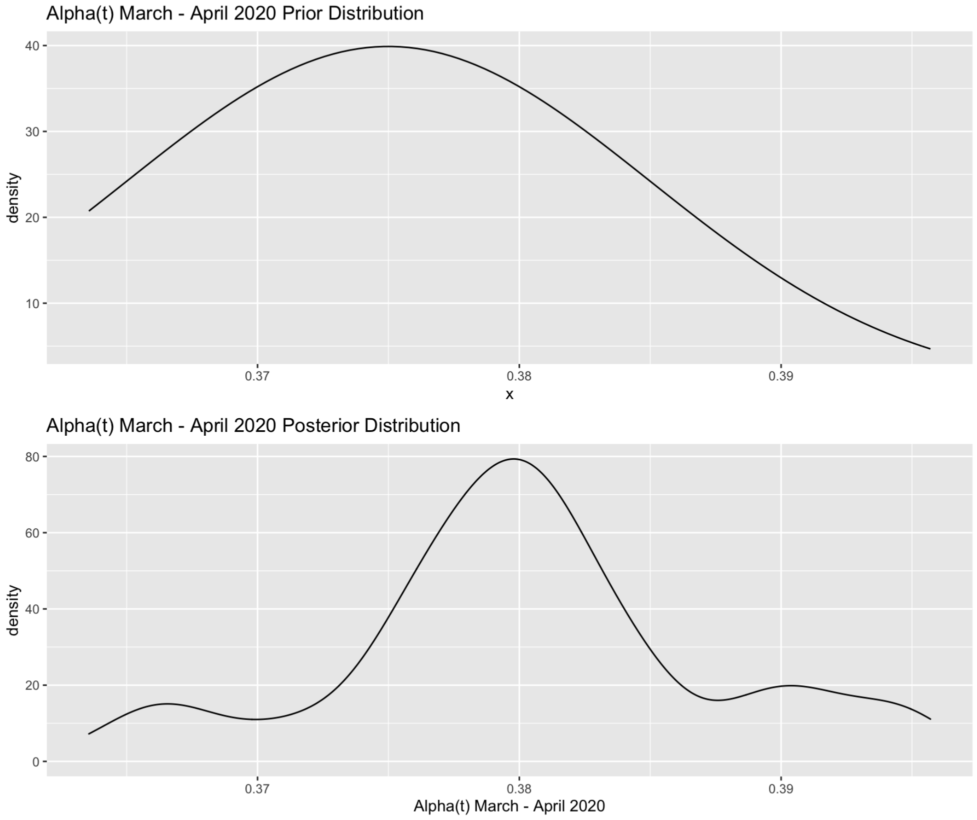

Figure 8. Probability of hospitalization given infection, α_t: value during March – April, 2020.

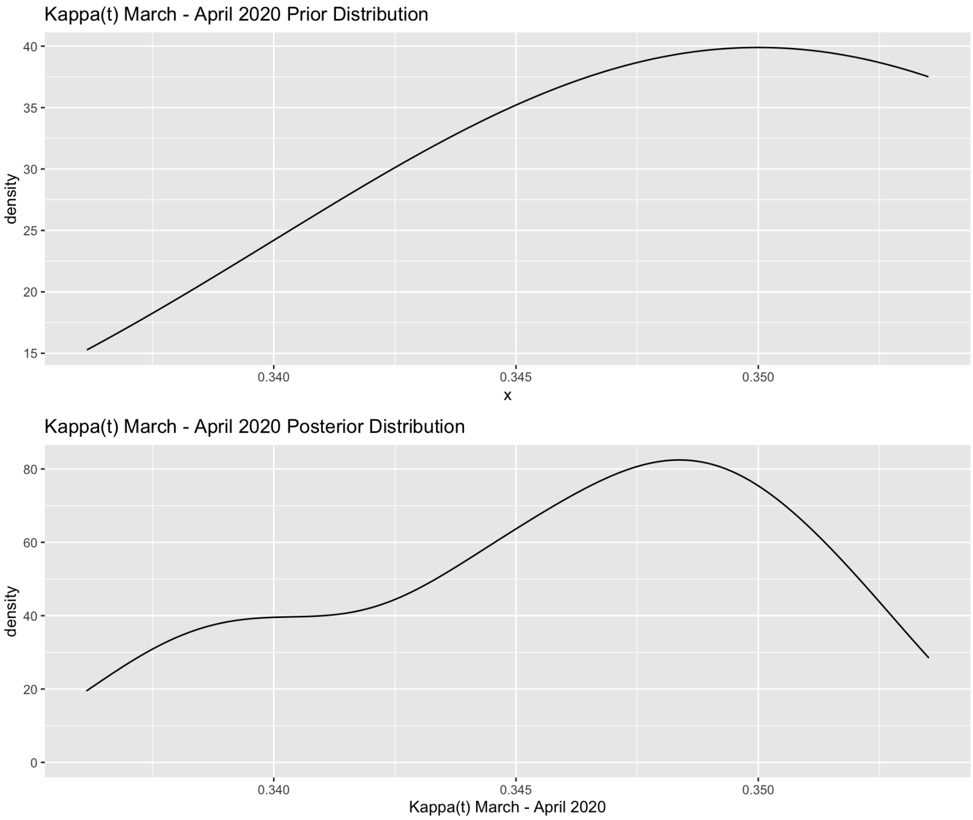

Figure 9. Probability of ICU admission given hospitalization, κ_t: value during March – April, 2020.

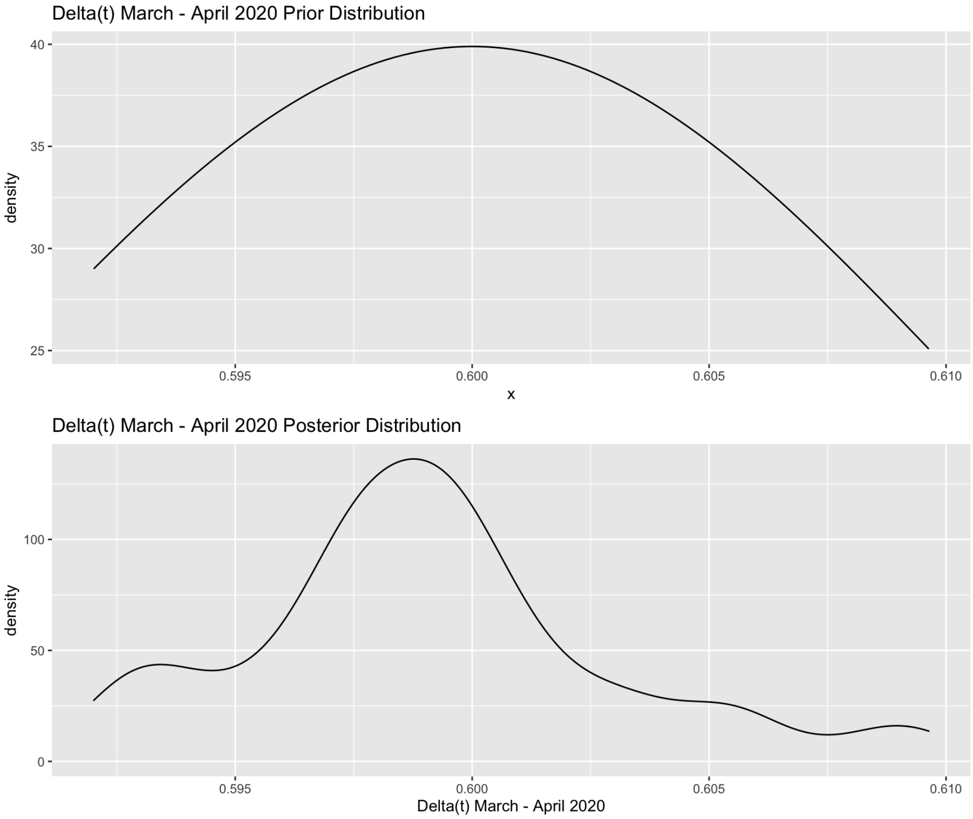

Figure 10. Probability of death given ICU admission, δ_t, during $t \in$ March – April, 2020.

Part II
Risk Model

This section provides details on the data inputs and calculations used in each of the 6 steps of the risk model described in the main text.

The notation used in this section is summarized in table 8 (in the order that they appear in the following text). Although not noted in the table, if a variable contains a subscript $_t$, this means it is time-varying.

Variable	Description	Type
$p \in P$	risk factor	definition
$q \in Q$	risk profile	definition
$m \in 1,2,3$	model for specific stage of disease progression	notation
m_1	model $(H\|I)$	definition
m_2	model $(Q\|H)$	definition
m_3	model $(D\|Q)$	definition
$\psi_{p,m}^{Marg}$	marginal log-RR vector for risk factor p and model m	data
ψ_{p}^{Cond}	conditional log-RR vector for risk factor p and model m	estimated by model
l_q	frequency vector of risk profile q in LAC	estimated by model
$\mathbf{l}_p$	marginal prevalence vector of risk factor p in LAC	data
Σ	reference correlation structure between risk factors p	calculated from data
$\mathbf{f}_{q,I}$	frequency vector of each risk profile in the infected population	estimated by model
p'	age group risk factor	definition
$f_{p',I_{obs}}$	observed frequency vector of each age group in the infected population	data
$\mathbf{R}$	risk profile design matrix (dimensions q x p)	definition
$\mathbf{R}_{p'}$	risk profile design matrix including only age categories (q x p')	definition
$\mathbf{l}_{p'}$	frequency vector of age group in LAC	estimated by model
$\widehat{P(H\|I)}$	probability vector for risk profiles for m_1	estimated by model
$\widehat{P(Q\|H)}$	probability vector for risk profiles for m_2	estimated by model
$\widehat{P(D\|Q)}$	probability vector for risk profiles for m_3	estimated by model
$\mathbf{X}_m$	mean-centered design matrix for model m (dimensions q x p)	estimated by model
$\mathbf{f}_{q,H}$	frequency vector of each risk profile in the hospitalized population	estimated by model
$\mathbf{f}_{q,Q}$	frequency vector of each risk profile in the in-ICU population	estimated by model
$\mathbf{f}_{q,D}$	frequency vector of each risk profile in the deceased population	estimated by model
CFR_q	vector of case fatality rates for each risk profile	estimated by model
IFR_q	vector of infection fatality rates for each risk profile	estimated by model

Table 8. Notation used within the risk model.

4 Modeled risk factors

The risk factors, $p \in P$, included in the risk model analysis are:

- **Age**
- **Body mass index (BMI)**
- **Smoking**
- **Any comorbidity**: diabetes, hypertension, chronic obstructive pulmonary disease (COPD), hepatitis B, coronary heart disease, stroke, cancer and chronic kidney disease.

Age was categorized within four groups: $0-18$, $19-49$, $50-64$, $65-79$, and 80+. We modeled BMI as an ordinal variable and assume an additive effect of BMI on the three models. BMI was categorized in three groups according to obesity classes: Class 1 (no obesity) $BMI < 30\frac{kg}{m^2}$; Class 2 (obesity), $30 \leq BMI \leq 40\frac{kg}{m^2}$; Class 3 (severe obesity), $BMI > 40\frac{kg}{m^2}$. Any comorbidity and smoking were modeled as binary variables. Note that risk factors are age, BMI, smoking and comorbidities, but age has 5 categories and BMI has 3 categories so $p = \{1,..,10\}$.

5 Step 2: Conditional RR for BMI, smoking, and comorbidities

We estimate the conditional relative risk (RR) effects corresponding to the risk factors BMI, smoking, and comorbidity conditional on age for each of the three risk models using marginal effects estimates available from reported studies and a method called the joint analysis of marginal summary statistics (JAM) [26]. JAM uses two pieces of information: (i) the marginal effect estimates between risk factors and outcomes for each model, and (ii) a reference correlation structure between the risk factors. For information informing (i) we obtain the log marginal RR between individual risk factors p and COVID-19 infection severity for each model m, $\psi_{p,m}^{Marg}$, from peer-reviewed clinical studies on patients with laboratory-confirmed COVID-19 [27, 28]. For (ii), we obtain the reference correlation structure, Σ, using data from The National Health and Nutrition Examination Survey (NHANES) from 2017-2018 [29].

5.1 Marginal relative risks between risk factors from published literature

We extracted the marginal RRs for each risk factor from clinical studies on patients with laboratory-confirmed COVID-19. Ordinal age, smoker, and any comorbidity we extracted from [27], a study reporting outcomes for 1099 patients from 552 hospitals in 30 provinces, autonomous regions, and municipalities in mainland China. The marginal RR of ordinal BMI were extracted from [28], with 4103 COVID-19 patients with laboratory-confirmed COVID-19 treated at a single academic health system in New York City. The left column of the main text Table 2 in the main text displays the marginal RRs extracted from the literature (95% confidence interval), $\psi_{p,m}^{Marg}$, for each risk factor and each of the three models hospitalization given illness, $(H|I)$, ICU admission given hospitalization, $(Q|H)$ and death given ICU admission, $(D|Q)$.

5.2 Correlation structure between risk factors

We obtain the correlation structure, Σ, between the risk factors p using data from The National Health and Nutrition Examination Survey (NHANES) [29]. NHANES is a survey research program conducted by the National Center for Health Statistics to assess the health and nutritional status of adults and children in the United States, and to track changes over time. We use the NHANES cohort of 2017-2018. To make the correlation matrix representative of the LAC population, we calculated it separately for each race/ethnicity and weight the correlation matrix by the distribution of the race/ethnicity in LAC.

5.3 Using JAM to estimate conditional RR

We use the JAM method [26] to calculate the conditional log relative risk (RR) for each risk factor, ψ_p^{Cond}, from the log of the marginal RR ψ_p^{Marg} and the correlation structure between risk factors Σ. The *reference group* was set as patients aged $0-18$, $BMI \leq 30\ \frac{kg}{m^2}$, non-smoker, and no comorbidities.

Using the marginal summary statistics from (i), specifically the marginal log relative risks $\psi_{p,m}^{Marg}$ for risk factor p and model m, JAM obtains conditional log relative risks $\psi_{p,m}^{Cond}$ for each factor. To accomplish this JAM first expresses the relationship between an outcome m, such as hospitalization given infection, ICU admission given hospitalization, and death given ICU admission, and the risk factors $p \in P$ as a normal linear model, $m \sim N(\mathbf{P}\psi, \tau^2\mathbf{I})$. For such a model the conditional or adjusted estimates of effect are given by $\hat{\psi} = (\mathbf{P}'\mathbf{P})^{-1}\mathbf{P}'\mathbf{m}$.

Heuristically, to fit this model without access to individual-level data we substitute an estimate of $\mathbf{P}'\mathbf{P}$ based on an estimate of this matrix using the correlation Σ between the risk factors from external NHANES data as specified in (ii). $\mathbf{P}'\mathbf{m}$ defines the mean value of the outcome for each of the corresponding values of the risk factor.

Technically, to adapt the linear model to use reference data, we first multiply all factors in the linear model by $\mathbf{P}'$ and define a P-length vector $\mathbf{z} := \mathbf{P}'\mathbf{m}$ to give $\mathbf{z} \sim N(\mathbf{P}'\mathbf{P}\psi, \tau^2\mathbf{P}'\mathbf{P})$. Since $\mathbf{P}'\mathbf{P}$ is inherently Hermitian and, if it is positive definite, we perform a Cholesky decomposition and simplify the likelihood by first defining $\mathbf{P}'\mathbf{P} = \mathbf{L}'\mathbf{L}$ and $\mathbf{zL} := \mathbf{L}'^{-1}\mathbf{z}$. Thus, we have $\mathbf{L}'^{-1}\mathbf{z} \sim MVN_P(\mathbf{L}\psi, \tau^2\mathbf{I_P})$. For this model the conditional estimates are then $\hat{\psi}_p{}^{Cond} = (\mathbf{L}'\mathbf{L})^{-1}\mathbf{z}$. $\mathbf{z}$ is constructed using marginal summary statistics and the frequencies for each risk factor. For a single binary risk factor, $\mathbf{z} := [(\hat{n}_0\hat{n}_1)/(\hat{n}_0+\hat{n}_1)\cdot\psi_p^{Marg}$, where $\hat{n}_0$ and $\hat{n}_1$ are the estimated counts of each group defined in the risk factor by $P = 0$ and $P = 1$, respectively. To get a plug-in for $\mathbf{P}'\mathbf{P}$ we use the following: $\mathbf{P}'\mathbf{P} \approx \mathbf{B}^{1/2}\Sigma\mathbf{B}^{1/2}$, where $\mathbf{B}$ gives the diagonal elements of $\mathbf{P}'\mathbf{P}$ defined using the frequencies of each risk factor and the sample size of the reference data. Σ is the estimated correlation matrix for the risk factors from the reference data. More details of the statistical methodology is in [26].

The resulting conditional relative risks (i.e., $\exp\hat{\psi}_{p,m}^{Cond}$) calculated by JAM for each risk factor and each of the three models are shown in the right column of Table 2 in the main text.

6 Step 3: Prevalence of each risk profile in the infected population

We estimate the time-varying frequency of each risk profile in the infected population, $\mathbf{f}_{t,q,I}$. First, we use available LAC data the prevalence of the individual risk factors within the overall LAC population [30] [31] to estimate the frequency of each risk profile within the overall population. Second, we use available data on the prevalence of each age group in illnesses [32]

together with our estimate of the prevalence of each risk factor in the overall LAC population to estimate the frequency of each profile within the infected population on each date. Illness timeseries data by age group is used because this is the only individual risk factor with observed infection prevalence data in LAC.

6.1 Frequency of risk profiles in overall LAC population

We estimate the frequency of the risk profiles q in the overall LAC population, l_q, by simulating a sample population based on the prevalence of each individual risk factor in LAC and the weighted correlation structure between the risk factors obtained from NHANES data, Σ (Section 5.2).

6.1.1 Data sources

The prevalence of age comes from the American Community Survey via the `tidycensus` R package [33]. The prevalence of obesity, comorbidities besides cancer, and smoking are taken from the Los Angeles County Health Survey (LACHS), study year 2018 [30]. To construct the obesity variable we find the $BMI \leq 30\frac{kg}{m^2}$ and $BMI > 30\frac{kg}{m^2}$ classes from LACHS. We then divide the $BMI > 30\frac{kg}{m^2}$ into the $30 \leq BMI < 40\frac{kg}{m^2}$ and $BMI > 40\frac{kg}{m^2}$ classes based on the relative ratios between these classes on average across the U.S., in data from the Behavioral Risk Factor Surveillance System study year 2010 [34]. The prevalence of cancer comes from the California Health Information Survey (CHIS) [31].

The prevalence of each of the 10 risk factors are combined in the vector $\mathbf{l}_p$.

6.1.2 Estimation

To calculate the frequency of the risk profiles q in the overall LAC population, l_q, we first generate a simulated population χ by sampling from a multivariate normal, $\chi \sim N(x; \mathbf{l}_p, \Sigma)$, where x is the number of samples, $\mathbf{l}_p$ is the vector of the prevalence of each individual risk factor in LAC, and Σ is the correlation structure between the risk factors as described in Section 5.2. Each sample from χ represents a specific risk profile, sampled in proportion to the probability of the combination of risk factors co-occurring. Prevalence of the *any comorbidity* category is constructed by sampling profiles expanded across all comorbidities, and creating an aggregated binary *any comorbidity* variable that takes value 1 if any of the comorbidities are included in the sampled profile.

We then calculate the vector of the frequencies of each risk profile q in the overall LAC population, $\mathbf{l}_q$, as its relative frequency in the simulated population χ.

6.2 Frequency of risk profiles in infected population

We obtain the frequency of each age group over illnesses, $f_{t,p',I_{obs}}$ from the LA Times data [21]. To estimate the frequency of each risk profile q in the infected population, $f_{t,q,I}$, the frequency of each age group over infections is stratified across the risk profiles according to the relative frequency of each profile within each age group in the overall LAC population.

We find $\mathbf{f}_{t,q,I}$, the vector of the frequency of each risk profile in the infected population, as follows.

6.2.1 Profile design matrix

First we define a risk profile design matrix $\mathbf{R}$, a q x p matrix, that includes indicator variables for the presence or absence of each risk factor p (columns) within each risk profile q (row), encoding the linear combination of all (plausible) linear combinations of the risk factors:

$$\mathbf{R} = \begin{bmatrix} R_{11} & \dots & R_{q1} \\ \dots & \dots & \dots \\ R_{1p} & \dots & R_{qp} \end{bmatrix}.$$

We also define $\mathbf{R}_{p'}$ to be a profile design matrix encoding age group risk factors only, i.e., a q x 5 matrix representing the first five columns of $\mathbf{R}$.

6.2.2 Calculation

We find the vector of the frequency of the risk profiles in the infected population, $\mathbf{f}_{t,q,I}$, using $\mathbf{l}_q$, $\mathbf{f}_{t,p',I_{obs}}$, and $\mathbf{R}_{p'}$.

First, let $\mathbf{l}_{p'}$ be a vector representing the frequency of each age group in the (estimated) overall population, found as $\mathbf{l}_{p'} = \mathbf{l}_q^\intercal \mathbf{R}_{p'}$.

Then we find $\mathbf{f}_{t,q,I}$ as

$$\mathbf{f}_{t,q,I} = \mathbf{l}_q \cdot \frac{\mathbf{R}_{p'}\mathbf{f}_{t,p',I_{obs}}}{\mathbf{R}_{p'}\mathbf{l}_{p'}}.$$

7 Step 4: Risk-profile-stratified probabilities of disease stage progression

7.1 Overview

We estimate the time-varying disease stage probabilities stratified across all risk profiles, $q \in Q$, for the three models $m = 1 : P_t(H|I)$, $m = 2 : P_t(Q|H)$, and $m = 3 : P_t(D|Q)$, resulting in the probability vectors $\underline{\widehat{P_t(H|I)}}$, $\underline{\widehat{P_t(Q|H)}}$, and $\underline{\widehat{P_t(H|I)}}$, respectively. This is done by combining in a logistic model all linear combinations of the p risk factors specified in a mean-centered design matrix, $\mathbf{X}_m$, and their corresponding conditional log-RR obtained from JAM, $\hat{\psi}^{Cond}_{p,m}$ (Section 5), with intercepts that are set to the logit of the estimated probabilities from the epidemic model for $\hat{\alpha}_t$, $\hat{\kappa}_t$, $\hat{\delta}_t$ (Section 2.1.4), respectively. The matrix $\mathbf{X}_m$ is a profile design matrix that has been mean-centered on the frequency of each risk profile in the incoming population relevant to each model m; namely, the infected (I) population for model 1, the hospitalized (H) population for model 2, and the in-ICU (Q) population for model 3. The specification of the frequency of each risk profile in the infected (I) population is described in Section 6. The frequency of each risk profile in the hospitalized population, $\mathbf{f}_{t,q,H}$ and the in-ICU population, $\mathbf{f}_{t,q,Q}$, are calculated recursively from the estimated frequency of each profile in the incoming population to each stage of disease, described in Section 7.2.3. Patients age $19-49$ with $BMI < 30\frac{kg}{m^2}$, non-smoker, and no comorbidities are the reference profile.

7.2 Inputs

7.2.1 Logit transformed population-average probabilities of each stage of disease $\hat{\alpha}_t$, $\hat{\kappa}_t$, $\hat{\delta}_t$

In Section 2.1.4 we estimate the population-average probability that individuals in LAC who acquire infection are admitted to hospital, $\hat{\alpha}_t$, who are in hospital require admittance to the ICU, $\hat{\kappa}_t$, and who are in ICU will die, $\hat{\delta}_t$. We logit-transform these probabilities as

$$
\begin{aligned}
\text{logit}(\hat{\alpha}_t) &= \log(\frac{\hat{\alpha}_t}{1-\hat{\alpha}_t}) \\
\text{logit}(\hat{\kappa}_t) &= \log(\frac{\hat{\kappa}_t}{1-\hat{\kappa}_t}) \\
\text{logit}(\hat{\delta}_t) &= \log(\frac{\hat{\delta}_t}{1-\hat{\delta}_t}).
\end{aligned}
$$

7.2.2 Vector of conditional log risk estimates for model m, $\underline{\hat{\psi}}^{Cond}_{m}$

In Section 5 we described the conditional log risk effects estimates, i.e., $\hat{\psi}^{Cond}_{p,m}$ for risk factor p and model m. We bring the conditional log risk effect estimates for model m together in to the vector $\underline{\hat{\psi}}^{Cond}_{m}$.

7.2.3 Frequencies of each incoming population $\mathbf{f}_{t,q,in}$

In Section 6 we estimated the frequency of each risk profile q in the infected population, $\mathbf{f}_{t,q,I}$ (a vector). The frequency of each risk profile in the hospitalized population, $\mathbf{f}_{t,q,H}$ and the in-ICU population, $\mathbf{f}_{t,q,Q}$, are calculated recursively from the estimated frequency of each profile in the incoming population to each stage of disease, as the normalized product of the frequency in the incoming stage of disease and the probability of advancing to the subsequent stage, normalized over all risk profiles.

Specifically, the frequency of each risk profile in the hospitalized population, $\mathbf{f}_{t,q,H}$, is calculated from the estimated $\underline{\widehat{P_t(H|I)}}$ and $\mathbf{f}_{t,q,I}$ as:

$$
\mathbf{f}_{t,q,H} = \frac{\widehat{\mathbf{P_t(H|I)}} \cdot \mathbf{f_{t,q,I}}}{\widehat{\mathbf{P_t(H|I)}}^{\mathsf{T}} \mathbf{f_{t,q,I}}},
$$

and the frequency of each risk profile in the in-ICU population, $\mathbf{f}_{t,q,Q}$, is calculated from the estimated $\widehat{P_t(Q|H)}$ and $\mathbf{f}_{t,q,H}$ as:

$$
\mathbf{f}_{t,q,Q} = \frac{\widehat{\mathbf{P_t(Q|H)}} \cdot \mathbf{f_{t,q,H}}}{\widehat{\mathbf{P_t(Q|H)}}^{\mathsf{T}} \mathbf{f_{t,q,H}}}.
$$

We also calculate the frequency of each profile in the deceased population, $\mathbf{f}_{t,q,D}$, which is needed in Step 5 and 6 although not for calculating the risk-profile-stratified probabilities of illness progression. $\mathbf{f}_{t,q,D}$ is found as the normalized product of the frequency in the incoming stage of disease, the in-ICU population, and the probability of advancing from the ICU to the deceased population, normalized over all risk profiles, i.e.:

$$
\mathbf{f}_{t,q,D} = \frac{\mathbf{Pr}\widehat{\mathbf{(D|Q)}}_{\mathbf{q,t}} \cdot \mathbf{f_{t,q,Q}}}{\mathbf{Pr}\widehat{\mathbf{(D|Q)}}_{\mathbf{q,t}}^{\mathsf{T}} \mathbf{f_{t,q,Q}}}.
$$

In the following we will require the marginal frequency of each risk factor p in each incoming population, $\mathbf{f}_{t,p,I}$, which we find by marginalizing the risk factors over the risk profiles q for each population as

$$\mathbf{f}_{t,p,in} = \mathbf{f}_{t,q,in}^{\mathsf{T}}\mathbf{R}. \tag{10}$$

7.2.4 Mean-centered design matrix $\mathbf{X}_m$, and risk profile design matrix $\mathbf{R}$

The mean-centered design matrix for model m, $\mathbf{X}_m$, is a profile design matrix that has been mean-centered on the frequency of each risk profile in the incoming population m_{in} relevant to each model m, $\mathbf{f}_{p,m_{in}}$; namely, the infected (I) population for model 1, the hospitalized (H) population for model 2, and the in-ICU (Q) population for model 3.

We find the mean-centered design matrices as:

$$\mathbf{X}_1 = \mathbf{R} - \mathbf{f}_{t,p,I}$$
$$\mathbf{X}_2 = \mathbf{R} - \mathbf{f}_{t,p,H}$$
$$\mathbf{X}_3 = \mathbf{R} - \mathbf{f}_{t,p,Q}.$$

7.3 Calculating risk-stratified probabilities

We find the probability of each risk profile for model 1, $\underline{\widehat{P_t(H|I)}}$, using the frequency of each risk factor in the infected population, $\mathbf{f}_{p,I}$, as:

$$\underline{\widehat{P_t(H|I)}} = \text{expit}(\text{logit}(\hat{\alpha}_t) + \mathbf{X_1}\underline{\hat{\psi}}_{m=1}^{Cond})$$

We can then find the probability of each risk profile for model 2, $\underline{\widehat{P_t(Q|H)}}$, using the frequency of each risk factor in the hospitalized population, $\mathbf{f}_{p,H}$, as:

$$\underline{\widehat{P_t(Q|H)}} = \text{expit}(\text{logit}(\hat{\kappa}_t) + \mathbf{X_2}\underline{\hat{\psi}}_{m=2}^{Cond}).$$

Finally, we find the probability of each risk profile for model 3, $\underline{\widehat{P_t(D|Q)}}$, using the frequency of each risk factor in the in-ICU population, $\mathbf{f}_{p,Q}$, as:

$$\underline{\widehat{P_t(D|Q)}} = \text{expit}(\text{logit}(\hat{\delta}_t) + \mathbf{X_3}\underline{\hat{\psi}}_{m=3}^{Cond}).$$

8 Step 5: Conditional RR for age

We estimate the conditional RR of each age group separately from that of the other three risk factors. This is done because while we can estimate the conditional RR using the same methodology as for the other factors as described in Step 2, we have observed data on the distribution of each age group over deaths for LAC that we can use to estimate the conditional RR for age, given the other model inputs and attributes. Given this information, we aim to find the solution set that minimizes the distance between the distribution over deaths produced by the logistic model and the observed distribution.

Specifically, we choose the conditional RR for age, $RR_{p'}$, such that the distance between the frequency of each age group over deaths produced in Step 4, $\mathbf{f}_{t,p',D}$ (marginalized from the profile-specific values $\mathbf{f}_{t,p',D}$ using equation 10) and the observed distribution of each age group over deaths in LAC, $\mathbf{f}_{t,p',D_{obs}}$, is minimized, i.e.

$$\arg\min_{RR_{p'}\in[0,10]} \text{Dist}_D = \sum_{t,p'} \mathbf{f}_{t,p',D}(RR_{p'}) - \mathbf{f}_{t,p',D_{obs}}, \tag{11}$$

where the frequency of each age group over deaths is written as $\mathbf{f}_{t,p',D}(RR_{p'})$ to emphasize that it is a function of the conditional RR of the age groups $RR_{p'}$.

This objective is solved by varying $RR_{p'}$ between $[0,10]$, rerunning Steps 3 and 4, and computing the distance metric Dist_D in equation 11.

9 Step 6: Risk-profile-stratified $CFR_{q,t}$ and $IFR_{q,t}$

We estimate the time-varying risk-profile-stratified case fatality rate (CFR_t) and the infection fatality rate (IFR(t)) from cumulative counts of observed infections, I_{cum}, total infections, $I_{cum} + A_{cum}$, and cumulative deaths D_{cum} coming from the epidemic model; and estimates of the time-varying frequency of each risk profile in the infected population, $\mathbf{f}_{t,q,I}$ (from Step 3) and of each risk profile in the deceased population (from Step 4).

We simulate model realizations with parameter values coming from this joint posterior distribution 2.3. For each realization, we estimate the number of individuals in each risk profile subpopulation in the observed infected population as the estimated frequency of each profile in the infected population, $\mathbf{f}_{t,q,I}$, multiplied by the epidemic-model-estimated time series of the cumulative number of observed infections (I_{cum}). Similarly, we estimate the number of individuals in each risk profile subpopulation in the total infected population (including observed an unobserved illnesses) as $\mathbf{f}_{t,q,I}$ multiplied by the estimated time series of cumulative total infections, i.e. $I_{cum} + A_{cum}$. We estimate the number of deceased individuals from each risk profile as $\mathbf{f}_{t,q,D}$ multiplied by the estimated time series of cumulative deaths (D_{cum}).

We find the CFR and IFR for each model realization as the estimated number of deaths for each profile over estimated observed infections for each profile, and number of deaths over total infections, respectively.

To produce uncertainty estimates, we aggregate model simulations from 100 jointly estimated parameter sets and 20 stochastic epidemic model realizations for each parameter set. We pool together all simulations and report their mean and 2.5th/97.5th percentiles.

10 Results for risk-profile-stratified estimates

10.1 Comparison between observed and estimated frequency of each age group in deceased population (for Step 5)

Figure 11 shows the frequency of the age groups in the deceased population estimated by the model, $\mathbf{f}_{t,p',D}(RR_{p'})$, compared with the observed frequency, $\mathbf{f}_{t,p',D_{obs}}$. Minimizing the summed differences between age groups p' in the distributions $\mathbf{f}_{t,p',D}(RR_{p'})$ and $\mathbf{f}_{t,p',D_{obs}}$, i.e. the metric Dist_D, is the objective used to estimate the conditional RR for age in Step 5 of the risk model methodology. Figure 11 demonstrates a very low difference error is achieved.

10.2 Profile-stratified probabilities of severe illness and death for LAC

The resulting probabilities across each risk profile for each of three models $\widehat{\underline{P_t}(H|I)}$, $\widehat{\underline{P_t}(Q|H)}$, and $\widehat{\underline{P_t}(D|Q)}$, as well as the estimated frequency of each profile in the overall LAC population across dates every two weeks from May 15 2020 - March 1 2021 are shown in Tables 9, 10, and 11, respectively.

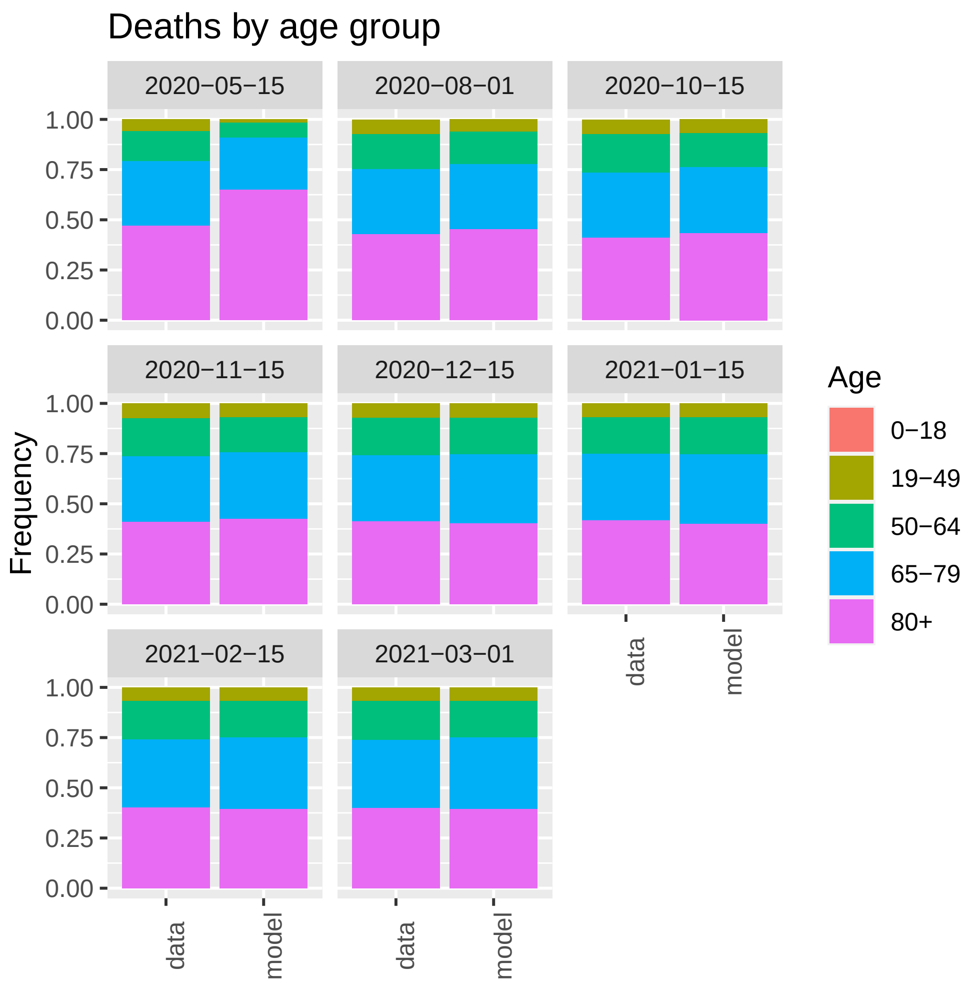

Figure 11. Frequency of the age groups in the deceased population estimated by the model, $\mathbf{f}_{t,p',D}(RR_{p'})$, compared with the observed frequency, $\mathbf{f}_{t,p',D_{obs}}$, used to estimate the conditional RR for age in Step 5 of the risk model.

Profile	Age	BMI	Smoker	Comorbidity	Prevalence	5/15/20	8/1/20	10/14/20	11/16/20	12/15/20	1/13/21	2/17/21	3/3/21
1	0-18	BMI<30	Smoker	No Comorbidity	0.02061	0.0139	0.0207	0.0134	0.0136	0.0139	0.014	0.0141	0.0141
2	0-18	BMI<30	Non Smoker	No Comorbidity	0.1517	0.0079	0.0118	0.0076	0.0077	0.0079	0.008	0.008	0.008
3	0-18	30<BMI<40	Smoker	No Comorbidity	0.00204	0.025	0.0371	0.0241	0.0244	0.025	0.0253	0.0254	0.0254
4	0-18	30<BMI<40	Non Smoker	No Comorbidity	0.03281	0.0143	0.0213	0.0138	0.014	0.0143	0.0145	0.0145	0.0145
5	0-18	BMI>40	Smoker	No Comorbidity	0.00045	0.0447	0.0655	0.0431	0.0436	0.0446	0.0452	0.0453	0.0453
6	0-18	BMI>40	Non Smoker	No Comorbidity	0.00674	0.0258	0.0382	0.0248	0.0252	0.0257	0.026	0.0261	0.0261
7	19-49	BMI<30	Non Smoker	No Comorbidity	0.16875	0.0556	0.0811	0.0537	0.0543	0.0555	0.0562	0.0564	0.0564
8	50-64	BMI<30	Non Smoker	No Comorbidity	0.04657	0.1321	0.1858	0.1279	0.1293	0.132	0.1334	0.1338	0.1338
9	65-79	BMI<30	Non Smoker	No Comorbidity	0.02863	0.2824	0.3712	0.2749	0.2775	0.2822	0.2847	0.2854	0.2855
10	80+	BMI<30	Non Smoker	No Comorbidity	0.02839	0.5169	0.616	0.5076	0.5108	0.5166	0.5196	0.5205	0.5206
11	19-49	30<BMI<40	Non Smoker	No Comorbidity	0.04291	0.0969	0.1386	0.0937	0.0948	0.0968	0.0978	0.0981	0.0982
12	50-64	30<BMI<40	Non Smoker	No Comorbidity	0.01836	0.2171	0.2937	0.2109	0.213	0.2169	0.219	0.2196	0.2197
13	65-79	30<BMI<40	Non Smoker	No Comorbidity	0.01088	0.4177	0.5182	0.4086	0.4117	0.4173	0.4203	0.4212	0.4213
14	80+	30<BMI<40	Non Smoker	No Comorbidity	0.00859	0.661	0.7451	0.6525	0.6554	0.6607	0.6634	0.6642	0.6643
15	19-49	BMI>40	Non Smoker	No Comorbidity	0.0101	0.1635	0.2267	0.1585	0.1602	0.1633	0.165	0.1655	0.1655
16	50-64	BMI>40	Non Smoker	No Comorbidity	0.00332	0.3357	0.4311	0.3274	0.3303	0.3354	0.3382	0.339	0.3391
17	65-79	BMI>40	Non Smoker	No Comorbidity	0.00155	0.5665	0.6621	0.5573	0.5605	0.5662	0.5692	0.57	0.5702
18	80+	BMI>40	Non Smoker	No Comorbidity	0.00124	0.7803	0.8419	0.7739	0.7761	0.7801	0.7822	0.7828	0.7829
19	19-49	BMI<30	Smoker	No Comorbidity	0.04085	0.0943	0.135	0.0911	0.0922	0.0942	0.0952	0.0955	0.0956
20	50-64	BMI<30	Smoker	No Comorbidity	0.00608	0.2121	0.2876	0.2059	0.208	0.2119	0.2139	0.2145	0.2146
21	65-79	BMI<30	Smoker	No Comorbidity	0.00148	0.4104	0.5107	0.4014	0.4045	0.4101	0.413	0.4139	0.414
22	80+	BMI<30	Smoker	No Comorbidity	0.00155	0.6542	0.7394	0.6457	0.6486	0.6539	0.6567	0.6575	0.6576
23	19-49	30<BMI<40	Smoker	No Comorbidity	0.00624	0.1594	0.2214	0.1545	0.1562	0.1593	0.1609	0.1614	0.1614
24	50-64	30<BMI<40	Smoker	No Comorbidity	0.00123	0.3291	0.4238	0.3209	0.3237	0.3288	0.3315	0.3323	0.3324
25	65-79	30<BMI<40	Smoker	No Comorbidity	0.00032	0.5591	0.6554	0.5499	0.5531	0.5588	0.5618	0.5627	0.5628
26	80+	30<BMI<40	Smoker	No Comorbidity	0.00016	0.7751	0.8379	0.7686	0.7708	0.7749	0.777	0.7777	0.7777
27	19-49	BMI>40	Smoker	No Comorbidity	0.0013	0.2568	0.3414	0.2498	0.2522	0.2566	0.2589	0.2596	0.2597
28	50-64	BMI>40	Smoker	No Comorbidity	0.00017	0.4719	0.5727	0.4626	0.4658	0.4716	0.4746	0.4755	0.4756
29	65-79	BMI>40	Smoker	No Comorbidity	0.00006	0.698	0.776	0.69	0.6928	0.6977	0.7003	0.701	0.7011
30	80+	BMI>40	Smoker	No Comorbidity	0.00002	0.8627	0.904	0.8582	0.8597	0.8625	0.864	0.8644	0.8644
31	19-49	BMI<30	Non Smoker	Comorbidity	0.10796	0.0815	0.1174	0.0787	0.0797	0.0814	0.0823	0.0826	0.0826
32	50-64	BMI<30	Non Smoker	Comorbidity	0.04482	0.1866	0.2559	0.181	0.1829	0.1864	0.1882	0.1888	0.1888
33	65-79	BMI<30	Non Smoker	Comorbidity	0.03342	0.3723	0.4707	0.3636	0.3666	0.372	0.3748	0.3757	0.3758
34	80+	BMI<30	Non Smoker	Comorbidity	0.02981	0.6172	0.7074	0.6083	0.6114	0.6169	0.6198	0.6206	0.6207
35	19-49	30<BMI<40	Non Smoker	Comorbidity	0.03314	0.1391	0.1951	0.1347	0.1362	0.139	0.1405	0.1409	0.1409
36	50-64	30<BMI<40	Non Smoker	Comorbidity	0.02078	0.2948	0.3853	0.2871	0.2897	0.2945	0.297	0.2978	0.2979
37	65-79	30<BMI<40	Non Smoker	Comorbidity	0.01545	0.5194	0.6184	0.5101	0.5133	0.5191	0.5221	0.523	0.5231
38	80+	30<BMI<40	Non Smoker	Comorbidity	0.00926	0.746	0.815	0.7389	0.7414	0.7458	0.7481	0.7488	0.7488
39	19-49	BMI>40	Non Smoker	Comorbidity	0.00804	0.2275	0.3063	0.221	0.2232	0.2273	0.2294	0.2301	0.2301
40	50-64	BMI>40	Non Smoker	Comorbidity	0.00378	0.4323	0.5331	0.4232	0.4263	0.432	0.435	0.4359	0.436
41	65-79	BMI>40	Non Smoker	Comorbidity	0.00218	0.6632	0.747	0.6548	0.6577	0.6629	0.6656	0.6664	0.6665
42	80+	BMI>40	Non Smoker	Comorbidity	0.00138	0.8426	0.8892	0.8376	0.8393	0.8424	0.844	0.8445	0.8445
43	19-49	BMI<30	Smoker	Comorbidity	0.02902	0.1356	0.1904	0.1313	0.1327	0.1354	0.1369	0.1373	0.1373
44	50-64	BMI<30	Smoker	Comorbidity	0.0063	0.2886	0.3782	0.281	0.2835	0.2883	0.2908	0.2915	0.2916
45	65-79	BMI<30	Smoker	Comorbidity	0.00138	0.5119	0.6113	0.5026	0.5058	0.5116	0.5146	0.5155	0.5156
46	80+	BMI<30	Smoker	Comorbidity	0.00182	0.7403	0.8104	0.7331	0.7356	0.7401	0.7424	0.7431	0.7432
47	19-49	30<BMI<40	Smoker	Comorbidity	0.00461	0.2223	0.3	0.2159	0.2181	0.2221	0.2242	0.2248	0.2249
48	50-64	30<BMI<40	Smoker	Comorbidity	0.00162	0.425	0.5257	0.4159	0.419	0.4247	0.4276	0.4285	0.4286
49	65-79	30<BMI<40	Smoker	Comorbidity	0.00039	0.6565	0.7413	0.648	0.6509	0.6562	0.6589	0.6597	0.6598
50	80+	30<BMI<40	Smoker	Comorbidity	0.00022	0.8386	0.8862	0.8334	0.8352	0.8384	0.84	0.8405	0.8406
51	19-49	BMI>40	Smoker	Comorbidity	0.00112	0.3424	0.4385	0.3341	0.3369	0.3422	0.3449	0.3457	0.3458
52	50-64	BMI>40	Smoker	Comorbidity	0.00026	0.5739	0.6688	0.5647	0.5678	0.5735	0.5765	0.5774	0.5775
53	65-79	BMI>40	Smoker	Comorbidity	0.00013	0.7769	0.8393	0.7703	0.7726	0.7767	0.7788	0.7794	0.7795
54	80+	BMI>40	Smoker	Comorbidity	0.00001	0.9044	0.9342	0.9012	0.9023	0.9043	0.9054	0.9057	0.9057

Table 9. Profile-stratified $\widehat{P_t(H|I)}$: Risk profiles (characterized by unique combination of age group, BMI range, smoking status, and any comorbidity), model-estimated population prevalence in LAC, and the probability of hospitalization given illness.

Profile	Age	BMI	Smoker	Comorbidity	Prevalence	5/15/20	8/1/20	10/14/20	11/16/20	12/15/20	1/13/21	2/17/21	3/3/21
1	0-18	BMI<30	Smoker	No Comorbidity	0.02061	0.0119	0.0189	0.0135	0.0136	0.0139	0.0138	0.0137	0.0137
2	0-18	BMI<30	Non Smoker	No Comorbidity	0.1517	0.0074	0.0118	0.0084	0.0085	0.0086	0.0086	0.0085	0.0085
3	0-18	30<BMI<40	Smoker	No Comorbidity	0.00204	0.0125	0.0198	0.0142	0.0143	0.0146	0.0145	0.0144	0.0144
4	0-18	30<BMI<40	Non Smoker	No Comorbidity	0.03281	0.0078	0.0123	0.0088	0.0089	0.0091	0.009	0.0089	0.0089
5	0-18	BMI>40	Smoker	No Comorbidity	0.00045	0.0131	0.0208	0.0149	0.015	0.0153	0.0152	0.0151	0.0151
6	0-18	BMI>40	Non Smoker	No Comorbidity	0.00674	0.0081	0.0129	0.0093	0.0093	0.0095	0.0095	0.0094	0.0094
7	19-49	BMI<30	Non Smoker	No Comorbidity	0.16875	0.0521	0.0808	0.0589	0.0594	0.0605	0.0602	0.0597	0.0596
8	50-64	BMI<30	Non Smoker	No Comorbidity	0.04657	0.1245	0.1852	0.1393	0.1404	0.1427	0.1421	0.1409	0.1409
9	65-79	BMI<30	Non Smoker	No Comorbidity	0.02863	0.2689	0.3701	0.2949	0.297	0.3009	0.2998	0.2978	0.2977
10	80+	BMI<30	Non Smoker	No Comorbidity	0.02839	0.4999	0.615	0.5321	0.5345	0.5392	0.5379	0.5355	0.5354
11	19-49	30<BMI<40	Non Smoker	No Comorbidity	0.04291	0.0546	0.0845	0.0617	0.0622	0.0633	0.063	0.0625	0.0624
12	50-64	30<BMI<40	Non Smoker	No Comorbidity	0.01836	0.13	0.1927	0.1452	0.1464	0.1488	0.1482	0.147	0.1469
13	65-79	30<BMI<40	Non Smoker	No Comorbidity	0.01088	0.2786	0.3816	0.3052	0.3073	0.3113	0.3102	0.3082	0.3081
14	80+	30<BMI<40	Non Smoker	No Comorbidity	0.00859	0.5122	0.6265	0.5443	0.5467	0.5513	0.55	0.5477	0.5476
15	19-49	BMI>40	Non Smoker	No Comorbidity	0.0101	0.0572	0.0884	0.0646	0.0651	0.0663	0.066	0.0654	0.0654
16	50-64	BMI>40	Non Smoker	No Comorbidity	0.00332	0.1356	0.2004	0.1514	0.1527	0.1551	0.1544	0.1532	0.1531
17	65-79	BMI>40	Non Smoker	No Comorbidity	0.00155	0.2886	0.3932	0.3157	0.3178	0.3219	0.3208	0.3187	0.3186
18	80+	BMI>40	Non Smoker	No Comorbidity	0.00124	0.5244	0.6379	0.5564	0.5588	0.5634	0.5621	0.5598	0.5597
19	19-49	BMI<30	Smoker	No Comorbidity	0.04085	0.0816	0.1244	0.0918	0.0926	0.0942	0.0938	0.093	0.093
20	50-64	BMI<30	Smoker	No Comorbidity	0.00608	0.1869	0.2686	0.2073	0.2089	0.212	0.2111	0.2096	0.2095
21	65-79	BMI<30	Smoker	No Comorbidity	0.00148	0.3728	0.4871	0.4034	0.4057	0.4103	0.409	0.4067	0.4066
22	80+	BMI<30	Smoker	No Comorbidity	0.00155	0.6177	0.7208	0.6476	0.6498	0.6541	0.6529	0.6508	0.6506
23	19-49	30<BMI<40	Smoker	No Comorbidity	0.00624	0.0854	0.1298	0.096	0.0968	0.0985	0.098	0.0972	0.0972
24	50-64	30<BMI<40	Smoker	No Comorbidity	0.00123	0.1945	0.2783	0.2154	0.2171	0.2203	0.2194	0.2178	0.2177
25	65-79	30<BMI<40	Smoker	No Comorbidity	0.00032	0.3843	0.4993	0.4152	0.4176	0.4222	0.4209	0.4186	0.4185
26	80+	30<BMI<40	Smoker	No Comorbidity	0.00016	0.6292	0.7305	0.6587	0.6609	0.6651	0.6639	0.6618	0.6617
27	19-49	BMI>40	Smoker	No Comorbidity	0.0013	0.0893	0.1354	0.1003	0.1012	0.1029	0.1025	0.1016	0.1015
28	50-64	BMI>40	Smoker	No Comorbidity	0.00017	0.2022	0.2883	0.2238	0.2255	0.2288	0.2279	0.2263	0.2262
29	65-79	BMI>40	Smoker	No Comorbidity	0.00006	0.396	0.5116	0.4271	0.4295	0.4342	0.4329	0.4306	0.4304
30	80+	BMI>40	Smoker	No Comorbidity	0.00002	0.6405	0.7401	0.6696	0.6718	0.6759	0.6748	0.6727	0.6726
31	19-49	BMI<30	Non Smoker	Comorbidity	0.10796	0.0531	0.0823	0.06	0.0606	0.0616	0.0613	0.0608	0.0608
32	50-64	BMI<30	Non Smoker	Comorbidity	0.04482	0.1267	0.1882	0.1417	0.1429	0.1452	0.1445	0.1434	0.1433
33	65-79	BMI<30	Non Smoker	Comorbidity	0.03342	0.2728	0.3748	0.2991	0.3012	0.3051	0.304	0.302	0.3019
34	80+	BMI<30	Non Smoker	Comorbidity	0.02981	0.5049	0.6197	0.5371	0.5395	0.5442	0.5429	0.5405	0.5404
35	19-49	30<BMI<40	Non Smoker	Comorbidity	0.03314	0.0557	0.0861	0.0628	0.0634	0.0645	0.0642	0.0636	0.0636
36	50-64	30<BMI<40	Non Smoker	Comorbidity	0.02078	0.1322	0.1958	0.1477	0.149	0.1514	0.1507	0.1495	0.1494
37	65-79	30<BMI<40	Non Smoker	Comorbidity	0.01545	0.2827	0.3863	0.3095	0.3116	0.3156	0.3145	0.3125	0.3124
38	80+	30<BMI<40	Non Smoker	Comorbidity	0.00926	0.5172	0.6312	0.5492	0.5516	0.5563	0.555	0.5527	0.5525
39	19-49	BMI>40	Non Smoker	Comorbidity	0.00804	0.0583	0.09	0.0658	0.0664	0.0676	0.0672	0.0666	0.0666
40	50-64	BMI>40	Non Smoker	Comorbidity	0.00378	0.138	0.2036	0.154	0.1553	0.1578	0.1571	0.1558	0.1558
41	65-79	BMI>40	Non Smoker	Comorbidity	0.00218	0.2927	0.398	0.3201	0.3222	0.3263	0.3252	0.3231	0.323
42	80+	BMI>40	Non Smoker	Comorbidity	0.00138	0.5294	0.6425	0.5613	0.5637	0.5683	0.5671	0.5647	0.5646
43	19-49	BMI<30	Smoker	Comorbidity	0.02902	0.0831	0.1266	0.0935	0.0943	0.096	0.0955	0.0947	0.0946
44	50-64	BMI<30	Smoker	Comorbidity	0.0063	0.19	0.2726	0.2106	0.2122	0.2154	0.2145	0.2129	0.2128
45	65-79	BMI<30	Smoker	Comorbidity	0.00138	0.3775	0.4921	0.4082	0.4105	0.4151	0.4138	0.4115	0.4114
46	80+	BMI<30	Smoker	Comorbidity	0.00182	0.6224	0.7248	0.6521	0.6544	0.6586	0.6574	0.6553	0.6552
47	19-49	30<BMI<40	Smoker	Comorbidity	0.00461	0.087	0.1321	0.0977	0.0986	0.1003	0.0998	0.099	0.0989
48	50-64	30<BMI<40	Smoker	Comorbidity	0.00162	0.1976	0.2824	0.2188	0.2205	0.2238	0.2228	0.2212	0.2211
49	65-79	30<BMI<40	Smoker	Comorbidity	0.00039	0.389	0.5043	0.4201	0.4224	0.427	0.4258	0.4235	0.4233

50	80+	30<BMI<40	Smoker	Comorbidity	0.00022	0.6338	0.7344	0.6632	0.6654	0.6695	0.6684	0.6663	0.6662
51	19-49	BMI>40	Smoker	Comorbidity	0.00112	0.0909	0.1378	0.1022	0.1031	0.1048	0.1043	0.1034	0.1034
52	50-64	BMI>40	Smoker	Comorbidity	0.00026	0.2055	0.2924	0.2273	0.229	0.2324	0.2315	0.2298	0.2297
53	65-79	BMI>40	Smoker	Comorbidity	0.00013	0.4008	0.5166	0.432	0.4344	0.4391	0.4378	0.4355	0.4353
54	80+	BMI>40	Smoker	Comorbidity	0.00001	0.6451	0.7439	0.674	0.6762	0.6803	0.6791	0.6771	0.677

Table 10. Profile-stratified $\widehat{P_t(Q|H)}$: Risk profiles (characterized by unique combination of age group, BMI range, smoking status, and any comorbidity), model-estimated population prevalence in LAC, and the probability of ICU admission given hospitalization.

Profile	Age	BMI	Smoker	Comorbidity	Prevalence	5/15/20	8/1/20	10/14/20	11/16/20	12/15/20	1/13/21	2/17/21	3/3/21
1	0-18	BMI<30	Smoker	No Comorbidity	0.02061	0.0305	0.0466	0.1027	0.104	0.1069	0.1067	0.1064	0.1064
2	0-18	BMI<30	Non Smoker	No Comorbidity	0.1517	0.0159	0.0244	0.0553	0.0561	0.0577	0.0576	0.0574	0.0574
3	0-18	30<BMI<40	Smoker	No Comorbidity	0.00204	0.034	0.0517	0.1133	0.1147	0.1178	0.1177	0.1173	0.1173
4	0-18	30<BMI<40	Non Smoker	No Comorbidity	0.03281	0.0177	0.0271	0.0614	0.0622	0.064	0.0639	0.0637	0.0637
5	0-18	BMI>40	Smoker	No Comorbidity	0.00045	0.0381	0.0579	0.1259	0.1275	0.1309	0.1307	0.1303	0.1303
6	0-18	BMI>40	Non Smoker	No Comorbidity	0.00674	0.0199	0.0305	0.0687	0.0696	0.0715	0.0715	0.0712	0.0712
7	19-49	BMI<30	Non Smoker	No Comorbidity	0.16875	0.1065	0.1559	0.3021	0.3051	0.3115	0.3112	0.3105	0.3104
8	50-64	BMI<30	Non Smoker	No Comorbidity	0.04657	0.2355	0.3231	0.5281	0.5316	0.5391	0.5388	0.5379	0.5379
9	65-79	BMI<30	Non Smoker	No Comorbidity	0.02863	0.4434	0.5525	0.7432	0.7459	0.7515	0.7513	0.7507	0.7506
10	80+	BMI<30	Non Smoker	No Comorbidity	0.02839	0.6841	0.7704	0.8872	0.8886	0.8916	0.8914	0.8911	0.8911
11	19-49	30<BMI<40	Non Smoker	No Comorbidity	0.04291	0.1174	0.1709	0.3258	0.3289	0.3355	0.3353	0.3345	0.3344
12	50-64	30<BMI<40	Non Smoker	No Comorbidity	0.01836	0.2559	0.3477	0.5554	0.5589	0.5663	0.566	0.5651	0.5651
13	65-79	30<BMI<40	Non Smoker	No Comorbidity	0.01088	0.4707	0.5795	0.7636	0.7662	0.7715	0.7713	0.7707	0.7706
14	80+	30<BMI<40	Non Smoker	No Comorbidity	0.00859	0.7074	0.7893	0.8978	0.8991	0.9017	0.9016	0.9013	0.9013
15	19-49	BMI>40	Non Smoker	No Comorbidity	0.0101	0.1304	0.1886	0.3526	0.3559	0.3628	0.3625	0.3617	0.3616
16	50-64	BMI>40	Non Smoker	No Comorbidity	0.00332	0.2794	0.3753	0.5848	0.5882	0.5955	0.5952	0.5944	0.5943
17	65-79	BMI>40	Non Smoker	No Comorbidity	0.00155	0.5006	0.6084	0.7846	0.787	0.792	0.7918	0.7912	0.7911
18	80+	BMI>40	Non Smoker	No Comorbidity	0.00124	0.7316	0.8086	0.9083	0.9094	0.9119	0.9118	0.9115	0.9115
19	19-49	BMI<30	Smoker	No Comorbidity	0.04085	0.1889	0.2651	0.4582	0.4618	0.4692	0.4689	0.4681	0.468
20	50-64	BMI<30	Smoker	No Comorbidity	0.00608	0.3758	0.4827	0.6862	0.6893	0.6957	0.6954	0.6947	0.6946
21	65-79	BMI<30	Smoker	No Comorbidity	0.00148	0.6089	0.707	0.8497	0.8515	0.8553	0.8551	0.8547	0.8547
22	80+	BMI<30	Smoker	No Comorbidity	0.00155	0.8089	0.8677	0.9389	0.9397	0.9414	0.9413	0.9411	0.9411
23	19-49	30<BMI<40	Smoker	No Comorbidity	0.00624	0.2063	0.2871	0.4856	0.4892	0.4967	0.4964	0.4955	0.4954
24	50-64	30<BMI<40	Smoker	No Comorbidity	0.00123	0.4019	0.5102	0.7094	0.7123	0.7184	0.7182	0.7175	0.7174
25	65-79	30<BMI<40	Smoker	No Comorbidity	0.00032	0.6347	0.7292	0.8632	0.8649	0.8684	0.8682	0.8679	0.8678
26	80+	30<BMI<40	Smoker	No Comorbidity	0.00016	0.8253	0.8798	0.9449	0.9457	0.9472	0.9471	0.947	0.9469
27	19-49	BMI>40	Smoker	No Comorbidity	0.0013	0.2266	0.3123	0.5156	0.5192	0.5267	0.5264	0.5255	0.5254
28	50-64	BMI>40	Smoker	No Comorbidity	0.00017	0.4311	0.5401	0.7335	0.7363	0.7421	0.7418	0.7412	0.7411
29	65-79	BMI>40	Smoker	No Comorbidity	0.00006	0.6621	0.7522	0.8768	0.8783	0.8815	0.8814	0.881	0.881
30	80+	BMI>40	Smoker	No Comorbidity	0.00002	0.8419	0.8919	0.9509	0.9515	0.9529	0.9528	0.9527	0.9527
31	19-49	BMI<30	Non Smoker	Comorbidity	0.10796	0.1103	0.1612	0.3106	0.3136	0.3201	0.3199	0.3191	0.319
32	50-64	BMI<30	Non Smoker	Comorbidity	0.04482	0.2428	0.332	0.5381	0.5416	0.549	0.5487	0.5479	0.5478
33	65-79	BMI<30	Non Smoker	Comorbidity	0.03342	0.4533	0.5623	0.7507	0.7534	0.7589	0.7587	0.7581	0.758
34	80+	BMI<30	Non Smoker	Comorbidity	0.02981	0.6927	0.7774	0.8911	0.8925	0.8954	0.8953	0.8949	0.8949
35	19-49	30<BMI<40	Non Smoker	Comorbidity	0.03314	0.1216	0.1766	0.3346	0.3378	0.3445	0.3442	0.3435	0.3434
36	50-64	30<BMI<40	Non Smoker	Comorbidity	0.02078	0.2636	0.3568	0.5653	0.5687	0.5761	0.5758	0.575	0.5749
37	65-79	30<BMI<40	Non Smoker	Comorbidity	0.01545	0.4807	0.5892	0.7707	0.7732	0.7785	0.7783	0.7777	0.7776
38	80+	30<BMI<40	Non Smoker	Comorbidity	0.00926	0.7156	0.7959	0.9014	0.9026	0.9052	0.9051	0.9048	0.9048
39	19-49	BMI>40	Non Smoker	Comorbidity	0.00804	0.135	0.1948	0.3618	0.3651	0.3721	0.3718	0.371	0.3709
40	50-64	BMI>40	Non Smoker	Comorbidity	0.00378	0.2875	0.3848	0.5945	0.5979	0.6051	0.6048	0.604	0.6039
41	65-79	BMI>40	Non Smoker	Comorbidity	0.00218	0.5106	0.6179	0.7913	0.7936	0.7985	0.7983	0.7977	0.7977
42	80+	BMI>40	Non Smoker	Comorbidity	0.00138	0.7393	0.8147	0.9115	0.9127	0.915	0.9149	0.9147	0.9146
43	19-49	BMI<30	Smoker	Comorbidity	0.02902	0.1951	0.273	0.4682	0.4717	0.4792	0.4789	0.478	0.4779
44	50-64	BMI<30	Smoker	Comorbidity	0.0063	0.3852	0.4927	0.6948	0.6978	0.7041	0.7038	0.7031	0.703

45	65-79	BMI<30	Smoker	Comorbidity	0.00138	0.6184	0.7152	0.8548	0.8565	0.8602	0.86	0.8596	0.8596
46	80+	BMI<30	Smoker	Comorbidity	0.00182	0.815	0.8722	0.9412	0.942	0.9436	0.9435	0.9433	0.9433
47	19-49	30<BMI<40	Smoker	Comorbidity	0.00461	0.2129	0.2954	0.4956	0.4992	0.5067	0.5064	0.5055	0.5054
48	50-64	30<BMI<40	Smoker	Comorbidity	0.00162	0.4116	0.5201	0.7176	0.7205	0.7265	0.7262	0.7255	0.7255
49	65-79	30<BMI<40	Smoker	Comorbidity	0.00039	0.644	0.737	0.8679	0.8695	0.8729	0.8728	0.8724	0.8723
50	80+	30<BMI<40	Smoker	Comorbidity	0.00022	0.831	0.884	0.947	0.9477	0.9492	0.9491	0.9489	0.9489
51	19-49	BMI>40	Smoker	Comorbidity	0.00112	0.2337	0.321	0.5256	0.5291	0.5366	0.5363	0.5355	0.5354
52	50-64	BMI>40	Smoker	Comorbidity	0.00026	0.4409	0.55	0.7413	0.744	0.7496	0.7494	0.7488	0.7487
53	65-79	BMI>40	Smoker	Comorbidity	0.00013	0.671	0.7596	0.8811	0.8825	0.8856	0.8855	0.8851	0.8851
54	80+	BMI>40	Smoker	Comorbidity	0.00001	0.8472	0.8957	0.9527	0.9533	0.9546	0.9546	0.9544	0.9544

Table 11. Profile-stratified $\widehat{P_t(D|Q)}$: Risk profiles (characterized by unique combination of age group, BMI range, smoking status, and any comorbidity), model-estimated population prevalence in LAC, and the probability of death given ICU admission.

10.3 CFR(t) and IFR(t) over all risk profiles

The median and 95% CI of resulting CFR(t) and IFR(t) across each risk profile, as well as the estimated frequency of each profile in the overall LAC population across dates every two weeks from May 15 2020 - March 1 2021 are shown in Tables 12 and 13.

ID	Age	BMI	Smoker	Comorbidity	Prev.	5/15/20	8/1/20	10/15/20	11/15/20	12/15/20	1/15/21	2/15/21	3/1/21
1	0-18	BMI<30	Smoker	No Comorbidity	0.02061	0.00001 (0.00001,0.00001)	0.00002 (0.00001,0.00003)	0.00002 (0.00002,0.00003)	0.00002 (0.00002,0.00003)	0.00002 (0.00001,0.00003)	0.00001 (0.00001,0.00002)	0.00001 (0.00001,0.00002)	0.00002 (0.00001,0.00002)
2	0-18	BMI<30	Non Smoker	No Comorbidity	0.1517	0.00001 (0.00001,0.00001)	0.00001 (0.00001,0.00001)	0.00001 (0.00001,0.00001)	0.00001 (0.00001,0.00001)	0.00001 (0,0.00001)	0.00001 (0,0.00001)	0.00001 (0,0.00001)	0.00001 (0,0.00001)
3	0-18	30<BMI<40	Smoker	No Comorbidity	0.00204	0.00003 (0.00002,0.00003)	0.00004 (0.00003,0.00006)	0.00005 (0.00004,0.00006)	0.00004 (0.00003,0.00006)	0.00003 (0.00002,0.00005)	0.00003 (0.00002,0.00005)	0.00003 (0.00002,0.00005)	0.00003 (0.00002,0.00005)
4	0-18	30<BMI<40	Non Smoker	No Comorbidity	0.03281	0.00001 (0.00001,0.00001)	0.00001 (0.00001,0.00001)	0.00001 (0.00001,0.00001)	0.00001 (0.00001,0.00001)	0.00001 (0,0.00001)	0.00001 (0,0.00001)	0.00001 (0,0.00001)	0.00001 (0,0.00001)
5	0-18	BMI>40	Smoker	No Comorbidity	0.00045	0.00006 (0.00005,0.00008)	0.00008 (0.00006,0.00012)	0.0001 (0.00008,0.00014)	0.00009 (0.00006,0.00012)	0.00007 (0.00005,0.00012)	0.00006 (0.00004,0.0001)	0.00006 (0.00004,0.00009)	0.00006 (0.00004,0.00009)
6	0-18	BMI>40	Non Smoker	No Comorbidity	0.00674	0.00001 (0.00001,0.00001)	0.00002 (0.00001,0.00002)	0.00002 (0.00001,0.00003)	0.00002 (0.00001,0.00002)	0.00001 (0.00001,0.00002)	0.00001 (0.00001,0.00002)	0.00001 (0.00001,0.00002)	0.00001 (0.00001,0.00002)
7	19-49	BMI<30	Non Smoker	No Comorbidity	0.16875	0.00062 (0.00053,0.00073)	0.00108 (0.00083,0.00151)	0.00117 (0.00089,0.00157)	0.00107 (0.00077,0.00146)	0.00084 (0.0005,0.00133)	0.00069 (0.00043,0.00115)	0.00076 (0.00052,0.00112)	0.00078 (0.00054,0.00113)
8	50-64	BMI<30	Non Smoker	No Comorbidity	0.04657	0.00778 (0.0067,0.00922)	0.01179 (0.00908,0.01641)	0.01157 (0.00877,0.01542)	0.01047 (0.00752,0.0143)	0.00813 (0.00489,0.01292)	0.00673 (0.00414,0.01113)	0.00734 (0.00504,0.01091)	0.0076 (0.00528,0.01099)
9	65-79	BMI<30	Non Smoker	No Comorbidity	0.02863	0.06758 (0.05823,0.08011)	0.08044 (0.06197,0.11202)	0.07416 (0.05623,0.09881)	0.06668 (0.04788,0.09103)	0.05112 (0.03076,0.08123)	0.04224 (0.026,0.06987)	0.04619 (0.03171,0.06864)	0.04784 (0.03321,0.06913)
10	80+	BMI<30	Non Smoker	No Comorbidity	0.02839	0.35477 (0.30571,0.42054)	0.30935 (0.23834,0.43079)	0.29487 (0.22355,0.39287)	0.26318 (0.18899,0.35929)	0.19892 (0.11969,0.31608)	0.16413 (0.10105,0.27151)	0.17984 (0.12347,0.26722)	0.18625 (0.12929,0.26914)
11	19-49	30<BMI<40	Non Smoker	No Comorbidity	0.04291	0.00125 (0.00107,0.00148)	0.00212 (0.00163,0.00295)	0.00232 (0.00176,0.00308)	0.0021 (0.00151,0.00287)	0.00165 (0.00099,0.00262)	0.00136 (0.00084,0.00225)	0.00148 (0.00102,0.00221)	0.00154 (0.00107,0.00222)
12	50-64	30<BMI<40	Non Smoker	No Comorbidity	0.01836	0.01449 (0.01249,0.01718)	0.02086 (0.01607,0.02904)	0.02093 (0.01587,0.02789)	0.01891 (0.01358,0.02582)	0.01464 (0.00881,0.02327)	0.0121 (0.00745,0.02001)	0.01321 (0.00907,0.01962)	0.01367 (0.00949,0.01976)
13	65-79	30<BMI<40	Non Smoker	No Comorbidity	0.01088	0.10992 (0.09472,0.1303)	0.12145 (0.09357,0.16913)	0.1172 (0.08886,0.15615)	0.10515 (0.07551,0.14356)	0.0803 (0.04832,0.12759)	0.06625 (0.04078,0.10959)	0.07242 (0.04972,0.10762)	0.075 (0.05206,0.10838)
14	80+	30<BMI<40	Non Smoker	No Comorbidity	0.00859	0.48059 (0.41412,0.56968)	0.39053 (0.30088,0.54384)	0.39238 (0.29748,0.52279)	0.34947 (0.25095,0.4771)	0.26312 (0.15832,0.4181)	0.21674 (0.13344,0.35854)	0.23739 (0.16298,0.35275)	0.24585 (0.17066,0.35527)
15	19-49	BMI>40	Non Smoker	No Comorbidity	0.0101	0.00245 (0.00211,0.0029)	0.004 (0.00308,0.00557)	0.00444 (0.00337,0.00591)	0.00403 (0.00289,0.0055)	0.00315 (0.00189,0.005)	0.0026 (0.0016,0.0043)	0.00283 (0.00195,0.00421)	0.00293 (0.00204,0.00424)
16	50-64	BMI>40	Non Smoker	No Comorbidity	0.00332	0.02553 (0.022,0.03026)	0.03437 (0.02648,0.04787)	0.03568 (0.02705,0.04754)	0.03218 (0.02311,0.04393)	0.02482 (0.01494,0.03944)	0.02048 (0.01261,0.03388)	0.02235 (0.01534,0.03321)	0.02314 (0.01606,0.03344)
17	65-79	BMI>40	Non Smoker	No Comorbidity	0.00155	0.16426 (0.14154,0.19471)	0.16791 (0.12936,0.23382)	0.16989 (0.1288,0.22636)	0.15208 (0.10921,0.20762)	0.11563 (0.06958,0.18374)	0.09523 (0.05863,0.15754)	0.10408 (0.07145,0.15465)	0.10777 (0.07481,0.15573)
18	80+	BMI>40	Non Smoker	No Comorbidity	0.00124	0.60082 (0.51773,0.7122)	0.46031 (0.35464,0.64101)	0.48123 (0.36484,0.64116)	0.42787 (0.30725,0.58413)	0.3211 (0.19321,0.51022)	0.26412 (0.16261,0.43693)	0.28922 (0.19856,0.42976)	0.29951 (0.20791,0.43281)
19	19-49	BMI<30	Smoker	No Comorbidity	0.04085	0.00292 (0.00251,0.00346)	0.00472 (0.00364,0.00657)	0.00472 (0.00358,0.00629)	0.00428 (0.00307,0.00584)	0.00334 (0.00201,0.0053)	0.00276 (0.0017,0.00456)	0.00301 (0.00207,0.00447)	0.00312 (0.00216,0.0045)
20	50-64	BMI<30	Smoker	No Comorbidity	0.00608	0.0299 (0.02576,0.03544)	0.03951 (0.03044,0.05503)	0.03604 (0.02732,0.04802)	0.03249 (0.02333,0.04435)	0.02503 (0.01506,0.03977)	0.02069 (0.01274,0.03423)	0.02261 (0.01552,0.03359)	0.02341 (0.01625,0.03383)
21	65-79	BMI<30	Smoker	No Comorbidity	0.00148	0.18694 (0.16109,0.2216)	0.18638 (0.14359,0.25954)	0.1693 (0.12835,0.22556)	0.15158 (0.10885,0.20694)	0.11526 (0.06935,0.18315)	0.09516 (0.05859,0.15742)	0.10417 (0.07152,0.15478)	0.10788 (0.07488,0.15589)
22	80+	BMI<30	Smoker	No Comorbidity	0.00155	0.656 (0.56528,0.77761)	0.49009 (0.37759,0.68248)	0.48316 (0.3663,0.64374)	0.42967 (0.30855,0.5866)	0.32254 (0.19408,0.51252)	0.26586 (0.16368,0.4398)	0.29153 (0.20015,0.43319)	0.30194 (0.20959,0.43631)
23	19-49	30<BMI<40	Smoker	No Comorbidity	0.00624	0.00564 (0.00486,0.00668)	0.00875 (0.00674,0.01218)	0.00886 (0.00672,0.01181)	0.00803 (0.00576,0.01096)	0.00624 (0.00376,0.00992)	0.00516 (0.00318,0.00853)	0.00563 (0.00386,0.00836)	0.00583 (0.00405,0.00842)
24	50-64	30<BMI<40	Smoker	No Comorbidity	0.00123	0.05162 (0.04448,0.06119)	0.06378 (0.04914,0.08882)	0.06035 (0.04575,0.08041)	0.0543 (0.03899,0.07413)	0.04169 (0.02508,0.06624)	0.03441 (0.02118,0.05692)	0.03759 (0.02581,0.05586)	0.03893 (0.02702,0.05625)
25	65-79	30<BMI<40	Smoker	No Comorbidity	0.00032	0.27371 (0.23585,0.32445)	0.2529 (0.19485,0.35218)	0.24256 (0.18389,0.32317)	0.21669 (0.15561,0.29583)	0.1641 (0.09874,0.26076)	0.13526 (0.08328,0.22376)	0.14796 (0.10158,0.21986)	0.15322 (0.10636,0.22141)
26	80+	30<BMI<40	Smoker	No Comorbidity	0.00016	0.80782 (0.6961,0.95758)	0.57065 (0.43965,0.79466)	0.58872 (0.44633,0.78438)	0.52224 (0.37502,0.71296)	0.3909 (0.23521,0.62114)	0.32174 (0.19808,0.53224)	0.3527 (0.24215,0.52409)	0.36529 (0.25356,0.52786)
27	19-49	BMI>40	Smoker	No Comorbidity	0.0013	0.01043 (0.00899,0.01237)	0.0153 (0.01179,0.02131)	0.0159 (0.01206,0.02119)	0.01438 (0.01032,0.01963)	0.01114 (0.00671,0.01771)	0.0092 (0.00566,0.01522)	0.01003 (0.00689,0.01491)	0.01039 (0.00721,0.01501)
28	50-64	BMI>40	Smoker	No Comorbidity	0.00017	0.08258 (0.07116,0.09789)	0.09452 (0.07282,0.13162)	0.09348 (0.07087,0.12454)	0.08391 (0.06026,0.11456)	0.06415 (0.0386,0.10194)	0.05287 (0.03255,0.08746)	0.05774 (0.03964,0.0858)	0.05979 (0.0415,0.08639)
29	65-79	BMI>40	Smoker	No Comorbidity	0.00006	0.36715 (0.31637,0.43521)	0.31631 (0.2437,0.44048)	0.31813 (0.24119,0.42386)	0.28362 (0.20367,0.3872)	0.21397 (0.12875,0.33999)	0.17612 (0.10843,0.29136)	0.19257 (0.13221,0.28614)	0.19943 (0.13843,0.28818)
30	80+	BMI>40	Smoker	No Comorbidity	0.00002	0.93201 (0.80312,1)	0.6363 (0.49023,0.88609)	0.66921 (0.50735,0.89162)	0.5978 (0.42928,0.81612)	0.44729 (0.26914,0.71074)	0.36766 (0.22635,0.60821)	0.40298 (0.27667,0.59881)	0.4175 (0.28981,0.6033)
31	19-49	BMI<30	Non Smoker	Comorbidity	0.10796	0.00096 (0.00083,0.00114)	0.00165 (0.00127,0.0023)	0.00181 (0.00137,0.00241)	0.00164 (0.00118,0.00224)	0.00129 (0.00077,0.00204)	0.00106 (0.00065,0.00176)	0.00116 (0.0008,0.00172)	0.0012 (0.00083,0.00174)
32	50-64	BMI<30	Non Smoker	Comorbidity	0.04482	0.01152 (0.00993,0.01366)	0.01695 (0.01306,0.0236)	0.01698 (0.01287,0.02262)	0.01535 (0.01102,0.02096)	0.0119 (0.00716,0.01891)	0.00983 (0.00605,0.01627)	0.01074 (0.00737,0.01595)	0.01112 (0.00772,0.01606)
33	65-79	BMI<30	Non Smoker	Comorbidity	0.03342	0.0924 (0.07962,0.10953)	0.10515 (0.08101,0.14643)	0.10048 (0.07618,0.13388)	0.09023 (0.06479,0.12318)	0.06901 (0.04152,0.10965)	0.05696 (0.03507,0.09423)	0.06228 (0.04276,0.09254)	0.06449 (0.04477,0.09319)
34	80+	BMI<30	Non Smoker	Comorbidity	0.02981	0.43321 (0.3733,0.51352)	0.36121 (0.27829,0.50301)	0.35829 (0.27164,0.47737)	0.31934 (0.22932,0.43597)	0.24076 (0.14487,0.38257)	0.19841 (0.12215,0.32823)	0.21734 (0.14921,0.32295)	0.22508 (0.15624,0.32526)
35	19-49	30<BMI<40	Non Smoker	Comorbidity	0.03314	0.00189 (0.00163,0.00224)	0.00314 (0.00242,0.00438)	0.00349 (0.00264,0.00464)	0.00316 (0.00227,0.00432)	0.00248 (0.00149,0.00393)	0.00205 (0.00126,0.00338)	0.00223 (0.00153,0.00331)	0.00231 (0.0016,0.00334)
36	50-64	30<BMI<40	Non Smoker	Comorbidity	0.02078	0.02062 (0.01777,0.02444)	0.02853 (0.02198,0.03973)	0.0295 (0.02236,0.0393)	0.02662 (0.01912,0.03635)	0.02057 (0.01238,0.03269)	0.01698 (0.01045,0.02809)	0.01853 (0.01272,0.02753)	0.01919 (0.01332,0.02772)
37	65-79	30<BMI<40	Non Smoker	Comorbidity	0.01545	0.14162 (0.12204,0.16788)	0.1492 (0.11495,0.20777)	0.14973 (0.11352,0.1995)	0.13415 (0.09633,0.18314)	0.10217 (0.06147,0.16234)	0.08418 (0.05183,0.13926)	0.09201 (0.06317,0.13672)	0.09528 (0.06614,0.13768)
38	80+	30<BMI<40	Non Smoker	Comorbidity	0.00926	0.5541 (0.47747,0.65682)	0.43392 (0.33431,0.60426)	0.45016 (0.34129,0.59978)	0.40045 (0.28756,0.54669)	0.30084 (0.18102,0.47803)	0.24756 (0.15241,0.40952)	0.27108 (0.18611,0.40281)	0.28073 (0.19487,0.40567)
39	19-49	BMI>40	Non Smoker	Comorbidity	0.00804	0.00359 (0.0031,0.00426)	0.00569 (0.00438,0.00792)	0.00647 (0.00491,0.00862)	0.00587 (0.00421,0.00801)	0.00458 (0.00275,0.00727)	0.00378 (0.00233,0.00625)	0.00412 (0.00283,0.00612)	0.00426 (0.00296,0.00616)
40	50-64	BMI>40	Non Smoker	Comorbidity	0.00378	0.03442 (0.02966,0.0408)	0.04428 (0.03411,0.06166)	0.04768 (0.03615,0.06353)	0.04294 (0.03083,0.05862)	0.03304 (0.01988,0.0525)	0.02722 (0.01676,0.04503)	0.0297 (0.02039,0.04413)	0.03075 (0.02135,0.04444)
41	65-79	BMI>40	Non Smoker	Comorbidity	0.00218	0.19894 (0.17143,0.23582)	0.19472 (0.15002,0.27116)	0.20408 (0.15472,0.2719)	0.18243 (0.131,0.24905)	0.13836 (0.08325,0.21985)	0.11382 (0.07007,0.18829)	0.12435 (0.08537,0.18478)	0.12876 (0.08938,0.18606)
42	80+	BMI>40	Non Smoker	Comorbidity	0.00138	0.66186 (0.57033,0.78456)	0.49331 (0.38007,0.68696)	0.5274 (0.39984,0.70267)	0.4684 (0.33636,0.63947)	0.35093 (0.21116,0.55763)	0.28846 (0.17759,0.47719)	0.31581 (0.21682,0.46927)	0.32704 (0.22702,0.47259)
43	19-49	BMI<30	Smoker	Comorbidity	0.02902	0.00441 (0.0038,0.00523)	0.00697 (0.00537,0.00971)	0.00707 (0.00536,0.00942)	0.00641 (0.0046,0.00875)	0.00499 (0.003,0.00793)	0.00412 (0.00254,0.00682)	0.0045 (0.00309,0.00669)	0.00466 (0.00323,0.00673)
44	50-64	BMI<30	Smoker	Comorbidity	0.0063	0.04238 (0.03652,0.05023)	0.05382 (0.04147,0.07495)	0.05058 (0.03835,0.06739)	0.04554 (0.03271,0.06218)	0.03502 (0.02107,0.05564)	0.02892 (0.0178,0.04784)	0.03159 (0.02169,0.04695)	0.03272 (0.02271,0.04728)
45	65-79	BMI<30	Smoker	Comorbidity	0.00138	0.23979 (0.20663,0.28425)	0.22801 (0.17567,0.31751)	0.21579 (0.1636,0.2875)	0.19293 (0.13854,0.26339)	0.14632 (0.08804,0.2325)	0.12065 (0.07428,0.19959)	0.13203 (0.09065,0.19619)	0.13673 (0.09491,0.19758)
46	80+	BMI<30	Smoker	Comorbidity	0.00182	0.75365 (0.64942,0.89336)	0.54302 (0.41836,0.75618)	0.55371 (0.41979,0.73773)	0.49184 (0.35319,0.67146)	0.36844 (0.22169,0.58544)	0.30338 (0.18678,0.50187)	0.33258 (0.22834,0.4942)	0.34445 (0.2391,0.49774)

47	19-49	30<BMI<40	Smoker	Comorbidity	0.00461	0.00826 (0.00712,0.00979)	0.01241 (0.00956,0.01728)	0.01287 (0.00976,0.01715)	0.01165 (0.00836,0.0159)	0.00904 (0.00544,0.01436)	0.00747 (0.0046,0.01235)	0.00814 (0.00559,0.0121)	0.00843 (0.00585,0.01218)
48	50-64	30<BMI<40	Smoker	Comorbidity	0.00162	0.06937 (0.05977,0.08223)	0.08183 (0.06305,0.11396)	0.08037 (0.06093,0.10708)	0.07221 (0.05185,0.09858)	0.0553 (0.03327,0.08786)	0.04559 (0.02807,0.07542)	0.04979 (0.03419,0.07399)	0.05156 (0.03579,0.07451)
49	65-79	30<BMI<40	Smoker	Comorbidity	0.00039	0.33008 (0.28443,0.39127)	0.29202 (0.22499,0.40666)	0.29076 (0.22044,0.3874)	0.2594 (0.18628,0.35414)	0.19596 (0.11791,0.31137)	0.16129 (0.0993,0.26682)	0.17644 (0.12114,0.26218)	0.18272 (0.12683,0.26404)
50	80+	30<BMI<40	Smoker	Comorbidity	0.00022	0.88641 (0.76382,1)	0.60997 (0.46995,0.84942)	0.64405 (0.48828,0.8581)	0.57142 (0.41033,0.78011)	0.42668 (0.25674,0.67799)	0.35094 (0.21606,0.58056)	0.38464 (0.26407,0.57154)	0.39835 (0.27652,0.57564)
51	19-49	BMI>40	Smoker	Comorbidity	0.00112	0.01461 (0.01259,0.01731)	0.02056 (0.01584,0.02862)	0.02208 (0.01674,0.02941)	0.01993 (0.01431,0.02721)	0.01542 (0.00928,0.02449)	0.01271 (0.00783,0.02103)	0.01386 (0.00952,0.0206)	0.01435 (0.00996,0.02074)
52	50-64	BMI>40	Smoker	Comorbidity	0.00026	0.10436 (0.08993,0.1237)	0.11399 (0.08782,0.15873)	0.11709 (0.08877,0.156)	0.10496 (0.07537,0.14329)	0.08003 (0.04816,0.12717)	0.06587 (0.04055,0.10897)	0.07191 (0.04937,0.10685)	0.07446 (0.05169,0.1076)
53	65-79	BMI>40	Smoker	Comorbidity	0.00013	0.41933 (0.36134,0.49707)	0.34891 (0.26882,0.48588)	0.36103 (0.27371,0.48101)	0.32149 (0.23086,0.43891)	0.24203 (0.14563,0.38458)	0.19888 (0.12244,0.32899)	0.21743 (0.14928,0.32308)	0.22515 (0.15629,0.32535)
54	80+	BMI>40	Smoker	Comorbidity	0.00001	0.98999 (0.85307,1)	0.66362 (0.51128,0.92413)	0.70896 (0.53749,0.94458)	0.62414 (0.4482,0.85209)	0.47262 (0.28438,0.75099)	0.38866 (0.23928,0.64295)	0.42604 (0.2925,0.63307)	0.44115 (0.30622,0.63748)

Table 12. Profile-stratified CFR(t) (as decimal). Risk profiles (characterized by unique combination of age group, BMI range, smoking status, and any comorbidity), model-estimated population prevalence in LAC, the frequency of each profile in the infection population, and the median and 95%CI of the Case Fatality Rate (CFR(t)).

ID	Age	BMI	Smoker	Comorbidity	Prev.	5/15/20	8/1/20	10/15/20	11/15/20	12/15/20	1/15/21	2/15/21	3/1/21
1	0-18	BMI<30	Smoker	No Comorbidity	0.02061	0.00001 (0,0.00001)	0.00001 (0,0.00001)	0.00001 (0,0.00002)	0.00001 (0,0.00001)	0.00001 (0,0.00001)	0.00001 (0,0.00001)	0.00001 (0,0.00001)	0.00001 (0,0.00001)
2	0-18	BMI<30	Non Smoker	No Comorbidity	0.1517	0.00001 (0,0.00001)	0.00001 (0,0.00001)	0.00001 (0,0.00002)	0.00001 (0,0.00001)	0.00001 (0,0.00001)	0.00001 (0,0.00001)	0.00001 (0,0.00001)	0.00001 (0,0.00001)
3	0-18	30<BMI<40	Smoker	No Comorbidity	0.00204	0.00001 (0,0.00001)	0.00001 (0,0.00001)	0.00001 (0,0.00002)	0.00001 (0,0.00001)	0.00001 (0,0.00001)	0.00001 (0,0.00001)	0.00001 (0,0.00001)	0.00001 (0,0.00001)
4	0-18	30<BMI<40	Non Smoker	No Comorbidity	0.03281	0.00001 (0,0.00001)	0.00001 (0,0.00001)	0.00001 (0,0.00002)	0.00001 (0,0.00001)	0.00001 (0,0.00001)	0.00001 (0,0.00001)	0.00001 (0,0.00001)	0.00001 (0,0.00001)
5	0-18	BMI>40	Smoker	No Comorbidity	0.00045	0.00001 (0.00001,0.00002)	0.00002 (0.00001,0.00003)	0.00002 (0.00001,0.00003)	0.00002 (0.00001,0.00003)	0.00001 (0.00001,0.00002)	0.00001 (0.00001,0.00002)	0.00001 (0.00001,0.00002)	0.00001 (0.00001,0.00002)
6	0-18	BMI>40	Non Smoker	No Comorbidity	0.00674	0.00001 (0,0.00001)	0.00001 (0,0.00001)	0.00001 (0,0.00002)	0.00001 (0,0.00001)	0.00001 (0,0.00001)	0.00001 (0,0.00001)	0.00001 (0,0.00001)	0.00001 (0,0.00001)
7	19-49	BMI<30	Non Smoker	No Comorbidity	0.16875	0.00011 (0.00006,0.00019)	0.0002 (0.0001,0.00036)	0.00021 (0.00011,0.00038)	0.00019 (0.0001,0.00034)	0.00015 (0.00008,0.00026)	0.00013 (0.00007,0.0002)	0.00014 (0.00008,0.00022)	0.00015 (0.00008,0.00023)
8	50-64	BMI<30	Non Smoker	No Comorbidity	0.04657	0.00144 (0.00075,0.00234)	0.00216 (0.00108,0.00388)	0.00211 (0.00107,0.00379)	0.00189 (0.00097,0.00336)	0.00147 (0.00077,0.00254)	0.00123 (0.00067,0.00195)	0.00137 (0.00075,0.00213)	0.00142 (0.00077,0.00225)
9	65-79	BMI<30	Non Smoker	No Comorbidity	0.02863	0.01248 (0.00648,0.02038)	0.01475 (0.00738,0.02648)	0.01354 (0.00683,0.02429)	0.01206 (0.00618,0.02142)	0.00926 (0.00487,0.01597)	0.00773 (0.00421,0.01225)	0.00861 (0.00471,0.0134)	0.00896 (0.00482,0.01413)
10	80+	BMI<30	Non Smoker	No Comorbidity	0.02839	0.0655 (0.034,0.10697)	0.05674 (0.02837,0.10186)	0.05384 (0.02716,0.09657)	0.04762 (0.0244,0.08455)	0.03605 (0.01895,0.06215)	0.03003 (0.01634,0.0476)	0.0335 (0.01833,0.05215)	0.03489 (0.01875,0.05503)
11	19-49	30<BMI<40	Non Smoker	No Comorbidity	0.04291	0.00023 (0.00012,0.00038)	0.00039 (0.00019,0.0007)	0.00042 (0.00021,0.00076)	0.00038 (0.0002,0.00068)	0.0003 (0.00016,0.00051)	0.00025 (0.00014,0.00039)	0.00028 (0.00015,0.00043)	0.00029 (0.00015,0.00045)
12	50-64	30<BMI<40	Non Smoker	No Comorbidity	0.01836	0.00268 (0.00139,0.00437)	0.00383 (0.00191,0.00687)	0.00382 (0.00193,0.00686)	0.00342 (0.00175,0.00608)	0.00265 (0.00139,0.00458)	0.00221 (0.0012,0.00351)	0.00246 (0.00135,0.00383)	0.00256 (0.00138,0.00404)
13	65-79	30<BMI<40	Non Smoker	No Comorbidity	0.01088	0.02029 (0.01054,0.03314)	0.02228 (0.01114,0.03999)	0.0214 (0.0108,0.03838)	0.01902 (0.00975,0.03378)	0.01455 (0.00765,0.02509)	0.01212 (0.0066,0.01921)	0.01349 (0.00738,0.021)	0.01405 (0.00755,0.02216)
14	80+	30<BMI<40	Non Smoker	No Comorbidity	0.00859	0.08872 (0.04606,0.14491)	0.07163 (0.03582,0.12858)	0.07164 (0.03615,0.12851)	0.06323 (0.0324,0.11227)	0.04768 (0.02506,0.0822)	0.03966 (0.02158,0.06286)	0.04422 (0.02419,0.06885)	0.04605 (0.02475,0.07264)
15	19-49	BMI>40	Non Smoker	No Comorbidity	0.0101	0.00045 (0.00023,0.00074)	0.00073 (0.00037,0.00132)	0.00081 (0.00041,0.00145)	0.00073 (0.00037,0.00129)	0.00057 (0.0003,0.00098)	0.00048 (0.00026,0.00075)	0.00053 (0.00029,0.00082)	0.00055 (0.0003,0.00087)
16	50-64	BMI>40	Non Smoker	No Comorbidity	0.00332	0.00471 (0.00245,0.0077)	0.0063 (0.00315,0.01132)	0.00652 (0.00329,0.01169)	0.00582 (0.00298,0.01034)	0.0045 (0.00236,0.00776)	0.00375 (0.00204,0.00594)	0.00416 (0.00228,0.00648)	0.00433 (0.00233,0.00684)
17	65-79	BMI>40	Non Smoker	No Comorbidity	0.00155	0.03032 (0.01574,0.04953)	0.0308 (0.0154,0.05528)	0.03102 (0.01565,0.05564)	0.02751 (0.0141,0.04886)	0.02095 (0.01101,0.03613)	0.01743 (0.00948,0.02762)	0.01939 (0.01061,0.03018)	0.02019 (0.01085,0.03184)
18	80+	BMI>40	Non Smoker	No Comorbidity	0.00124	0.11092 (0.05758,0.18116)	0.08443 (0.04222,0.15156)	0.08786 (0.04433,0.1576)	0.07741 (0.03966,0.13746)	0.05819 (0.03058,0.10032)	0.04833 (0.0263,0.0766)	0.05388 (0.02947,0.08388)	0.05611 (0.03015,0.0885)
19	19-49	BMI<30	Smoker	No Comorbidity	0.04085	0.00054 (0.00028,0.00088)	0.00087 (0.00043,0.00155)	0.00086 (0.00043,0.00155)	0.00077 (0.0004,0.00137)	0.0006 (0.00032,0.00104)	0.0005 (0.00027,0.0008)	0.00056 (0.00031,0.00087)	0.00058 (0.00031,0.00092)
20	50-64	BMI<30	Smoker	No Comorbidity	0.00608	0.00552 (0.00287,0.00901)	0.00725 (0.00362,0.01301)	0.00658 (0.00332,0.0118)	0.00588 (0.00301,0.01044)	0.00454 (0.00238,0.00782)	0.00379 (0.00206,0.006)	0.00421 (0.0023,0.00656)	0.00439 (0.00236,0.00692)
21	65-79	BMI<30	Smoker	No Comorbidity	0.00148	0.03451 (0.01792,0.05637)	0.03418 (0.01709,0.06136)	0.03091 (0.0156,0.05545)	0.02742 (0.01405,0.0487)	0.02089 (0.01098,0.03601)	0.01741 (0.00948,0.0276)	0.0194 (0.01061,0.03021)	0.02021 (0.01086,0.03187)
22	80+	BMI<30	Smoker	No Comorbidity	0.00155	0.12111 (0.06287,0.1978)	0.08989 (0.04495,0.16136)	0.08821 (0.04451,0.15824)	0.07774 (0.03983,0.13804)	0.05845 (0.03072,0.10077)	0.04865 (0.02647,0.0771)	0.05431 (0.02971,0.08455)	0.05656 (0.0304,0.08921)
23	19-49	30<BMI<40	Smoker	No Comorbidity	0.00624	0.00104 (0.00054,0.0017)	0.0016 (0.0008,0.00288)	0.00162 (0.00082,0.0029)	0.00145 (0.00074,0.00258)	0.00113 (0.00059,0.00195)	0.00094 (0.00051,0.0015)	0.00105 (0.00057,0.00163)	0.00109 (0.00059,0.00172)
24	50-64	30<BMI<40	Smoker	No Comorbidity	0.00123	0.00953 (0.00495,0.01556)	0.0117 (0.00585,0.021)	0.01102 (0.00556,0.01977)	0.00982 (0.00503,0.01744)	0.00755 (0.00397,0.01302)	0.0063 (0.00343,0.00998)	0.007 (0.00383,0.0109)	0.00729 (0.00392,0.0115)
25	65-79	30<BMI<40	Smoker	No Comorbidity	0.00032	0.05053 (0.02623,0.08253)	0.04639 (0.02319,0.08327)	0.04428 (0.02234,0.07944)	0.03921 (0.02009,0.06962)	0.02974 (0.01563,0.05127)	0.02475 (0.01347,0.03923)	0.02756 (0.01508,0.04291)	0.0287 (0.01543,0.04527)
26	80+	30<BMI<40	Smoker	No Comorbidity	0.00016	0.14913 (0.07742,0.24357)	0.10467 (0.05233,0.18789)	0.10749 (0.05423,0.19281)	0.09448 (0.04841,0.16777)	0.07083 (0.03723,0.12212)	0.05887 (0.03204,0.09331)	0.0657 (0.03594,0.10229)	0.06843 (0.03678,0.10793)
27	19-49	BMI>40	Smoker	No Comorbidity	0.0013	0.00193 (0.001,0.00315)	0.00281 (0.0014,0.00504)	0.0029 (0.00147,0.00521)	0.0026 (0.00133,0.00462)	0.00202 (0.00106,0.00348)	0.00168 (0.00092,0.00267)	0.00187 (0.00102,0.00291)	0.00195 (0.00105,0.00307)
28	50-64	BMI>40	Smoker	No Comorbidity	0.00017	0.01525 (0.00791,0.0249)	0.01734 (0.00867,0.03112)	0.01707 (0.00861,0.03061)	0.01518 (0.00778,0.02696)	0.01162 (0.00611,0.02004)	0.00967 (0.00526,0.01533)	0.01076 (0.00588,0.01674)	0.0112 (0.00602,0.01766)
29	65-79	BMI>40	Smoker	No Comorbidity	0.00006	0.06778 (0.03519,0.1107)	0.05802 (0.02901,0.10415)	0.05808 (0.02931,0.10419)	0.05131 (0.02629,0.09112)	0.03877 (0.02038,0.06685)	0.03223 (0.01754,0.05108)	0.03587 (0.01962,0.05585)	0.03736 (0.02008,0.05892)
30	80+	BMI>40	Smoker	No Comorbidity	0.00002	0.17206 (0.08932,0.28102)	0.11671 (0.05836,0.2095)	0.12218 (0.06165,0.21917)	0.10816 (0.05542,0.19205)	0.08105 (0.0426,0.13974)	0.06728 (0.03661,0.10663)	0.07507 (0.04107,0.11687)	0.07821 (0.04203,0.12336)
31	19-49	BMI<30	Non Smoker	Comorbidity	0.10796	0.00018 (0.00009,0.00029)	0.0003 (0.00015,0.00054)	0.00033 (0.00017,0.00059)	0.0003 (0.00015,0.00053)	0.00023 (0.00012,0.0004)	0.00019 (0.00011,0.00031)	0.00022 (0.00012,0.00034)	0.00022 (0.00012,0.00035)
32	50-64	BMI<30	Non Smoker	Comorbidity	0.04482	0.00213 (0.0011,0.00347)	0.00311 (0.00155,0.00558)	0.0031 (0.00156,0.00556)	0.00278 (0.00142,0.00493)	0.00216 (0.00113,0.00372)	0.0018 (0.00098,0.00285)	0.002 (0.00109,0.00311)	0.00208 (0.00112,0.00328)
33	65-79	BMI<30	Non Smoker	Comorbidity	0.03342	0.01706 (0.00886,0.02786)	0.01929 (0.00964,0.03462)	0.01835 (0.00926,0.03291)	0.01632 (0.00836,0.02899)	0.0125 (0.00657,0.02156)	0.01042 (0.00567,0.01652)	0.0116 (0.00635,0.01806)	0.01208 (0.00649,0.01905)
34	80+	BMI<30	Non Smoker	Comorbidity	0.02981	0.07998 (0.04152,0.13062)	0.06625 (0.03313,0.11893)	0.06542 (0.03301,0.11734)	0.05778 (0.0296,0.10259)	0.04363 (0.02293,0.07522)	0.03631 (0.01976,0.05754)	0.04049 (0.02215,0.06303)	0.04216 (0.02266,0.0665)
35	19-49	30<BMI<40	Non Smoker	Comorbidity	0.03314	0.00035 (0.00018,0.00057)	0.00058 (0.00029,0.00103)	0.00064 (0.00032,0.00114)	0.00057 (0.00029,0.00102)	0.00045 (0.00024,0.00077)	0.00037 (0.0002,0.00059)	0.00042 (0.00023,0.00065)	0.00043 (0.00023,0.00068)
36	50-64	30<BMI<40	Non Smoker	Comorbidity	0.02078	0.00381 (0.00198,0.00622)	0.00523 (0.00262,0.00939)	0.00539 (0.00272,0.00966)	0.00482 (0.00247,0.00855)	0.00373 (0.00196,0.00643)	0.00311 (0.00169,0.00492)	0.00345 (0.00189,0.00537)	0.00359 (0.00193,0.00567)
37	65-79	30<BMI<40	Non Smoker	Comorbidity	0.01545	0.02614 (0.01357,0.0427)	0.02737 (0.01368,0.04912)	0.02734 (0.01379,0.04904)	0.02427 (0.01244,0.0431)	0.01851 (0.00973,0.03192)	0.0154 (0.00838,0.02442)	0.01714 (0.00938,0.02668)	0.01785 (0.00959,0.02815)
38	80+	30<BMI<40	Non Smoker	Comorbidity	0.00926	0.10229 (0.05311,0.16707)	0.07959 (0.03979,0.14287)	0.08219 (0.04147,0.14743)	0.07245 (0.03712,0.12865)	0.05452 (0.02865,0.09399)	0.0453 (0.02465,0.0718)	0.0505 (0.02762,0.07862)	0.05259 (0.02826,0.08295)
39	19-49	BMI>40	Non Smoker	Comorbidity	0.00804	0.00066 (0.00034,0.00108)	0.00104 (0.00052,0.00187)	0.00118 (0.0006,0.00212)	0.00106 (0.00054,0.00189)	0.00083 (0.00044,0.00143)	0.00069 (0.00038,0.0011)	0.00077 (0.00042,0.00119)	0.0008 (0.00043,0.00126)
40	50-64	BMI>40	Non Smoker	Comorbidity	0.00378	0.00635 (0.0033,0.01038)	0.00812 (0.00406,0.01458)	0.00871 (0.00439,0.01562)	0.00777 (0.00398,0.01379)	0.00599 (0.00315,0.01032)	0.00498 (0.00271,0.0079)	0.00553 (0.00303,0.00861)	0.00576 (0.0031,0.00909)
41	65-79	BMI>40	Non Smoker	Comorbidity	0.00218	0.03673 (0.01907,0.05998)	0.03572 (0.01786,0.06411)	0.03726 (0.0188,0.06684)	0.03301 (0.01691,0.05861)	0.02507 (0.01318,0.04323)	0.02083 (0.01133,0.03301)	0.02317 (0.01267,0.03606)	0.02412 (0.01296,0.03804)
42	80+	BMI>40	Non Smoker	Comorbidity	0.00138	0.12219 (0.06343,0.19956)	0.09048 (0.04524,0.16242)	0.09629 (0.04858,0.17272)	0.08474 (0.04342,0.15048)	0.06359 (0.03342,0.10964)	0.05278 (0.02872,0.08366)	0.05883 (0.03218,0.09159)	0.06126 (0.03293,0.09663)
43	19-49	BMI<30	Smoker	Comorbidity	0.02902	0.00081 (0.00042,0.00133)	0.00128 (0.00064,0.0023)	0.00129 (0.00065,0.00232)	0.00116 (0.00059,0.00206)	0.0009 (0.00048,0.00156)	0.00075 (0.00041,0.0012)	0.00084 (0.00046,0.0013)	0.00087 (0.00047,0.00138)
44	50-64	BMI<30	Smoker	Comorbidity	0.0063	0.00782 (0.00406,0.01278)	0.00987 (0.00494,0.01772)	0.00924 (0.00466,0.01657)	0.00824 (0.00422,0.01463)	0.00635 (0.00334,0.01094)	0.00529 (0.00288,0.00839)	0.00589 (0.00322,0.00916)	0.00613 (0.00329,0.00967)
45	65-79	BMI<30	Smoker	Comorbidity	0.00138	0.04427 (0.02298,0.0723)	0.04182 (0.02091,0.07507)	0.0394 (0.01988,0.07067)	0.03491 (0.01788,0.06198)	0.02651 (0.01394,0.04571)	0.02208 (0.01201,0.03499)	0.0246 (0.01345,0.03829)	0.02561 (0.01377,0.0404)
46	80+	BMI<30	Smoker	Comorbidity	0.00182	0.13913 (0.07223,0.22724)	0.0996 (0.0498,0.17879)	0.10109 (0.05101,0.18134)	0.08898 (0.04559,0.15801)	0.06676 (0.03509,0.11511)	0.05551 (0.03021,0.08799)	0.06196 (0.03389,0.09645)	0.06452 (0.03468,0.10177)
47	19-49	30<BMI<40	Smoker	Comorbidity	0.00461	0.00152 (0.00079,0.00249)	0.00228 (0.00114,0.00408)	0.00235 (0.00119,0.00422)	0.00211 (0.00108,0.00374)	0.00164 (0.00086,0.00282)	0.00137 (0.00074,0.00217)	0.00152 (0.00083,0.00236)	0.00158 (0.00085,0.00249)
48	50-64	30<BMI<40	Smoker	Comorbidity	0.00162	0.01281 (0.00665,0.02092)	0.01501 (0.0075,0.02694)	0.01467 (0.0074,0.02632)	0.01306 (0.00669,0.0232)	0.01002 (0.00527,0.01728)	0.00834 (0.00454,0.01322)	0.00928 (0.00507,0.01444)	0.00966 (0.00519,0.01523)
49	65-79	30<BMI<40	Smoker	Comorbidity	0.00039	0.06094 (0.03164,0.09953)	0.05356 (0.02678,0.09615)	0.05309 (0.02679,0.09523)	0.04693 (0.02405,0.08334)	0.03551 (0.01866,0.06122)	0.02951 (0.01606,0.04678)	0.03287 (0.01798,0.05117)	0.03423 (0.0184,0.05399)
50	80+	30<BMI<40	Smoker	Comorbidity	0.00022	0.16364 (0.08495,0.26727)	0.11188 (0.05594,0.20083)	0.11759 (0.05933,0.21093)	0.10338 (0.05297,0.18358)	0.07732 (0.04064,0.1333)	0.06422 (0.03495,0.10178)	0.07165 (0.0392,0.11155)	0.07462 (0.04011,0.1177)
51	19-49	BMI>40	Smoker	Comorbidity	0.00112	0.0027 (0.0014,0.0044)	0.00377 (0.00189,0.00677)	0.00403 (0.00203,0.00723)	0.00361 (0.00185,0.0064)	0.00279 (0.00147,0.00482)	0.00233 (0.00127,0.00369)	0.00258 (0.00141,0.00402)	0.00269 (0.00145,0.00424)
52	50-64	BMI>40	Smoker	Comorbidity	0.00026	0.01927 (0.01,0.03147)	0.02091 (0.01045,0.03753)	0.02138 (0.01079,0.03835)	0.01899 (0.00973,0.03372)	0.0145 (0.00762,0.025)	0.01205 (0.00656,0.0191)	0.0134 (0.00733,0.02085)	0.01395 (0.0075,0.022)
53	65-79	BMI>40	Smoker	Comorbidity	0.00013	0.07741 (0.04019,0.12644)	0.064 (0.032,0.11488)	0.06591 (0.03326,0.11824)	0.05817 (0.0298,0.10328)	0.04386 (0.02305,0.07562)	0.03639 (0.0198,0.05768)	0.0405 (0.02216,0.06306)	0.04218 (0.02267,0.06652)
54	80+	BMI>40	Smoker	Comorbidity	0.00001	0.18276 (0.09488,0.2985)	0.12172 (0.06086,0.2185)	0.12944 (0.06531,0.23219)	0.11292 (0.05786,0.20051)	0.08564 (0.04501,0.14766)	0.07112 (0.0387,0.11272)	0.07937 (0.04342,0.12356)	0.08264 (0.04441,0.13034)

Table 13. Profile-stratified IFR(t) (as decimal). Risk profiles (characterized by unique combination of age group, BMI range, smoking status, and any comorbidity), model-estimated population prevalence in LAC, the frequency of each profile in the infection population, and the median and 95%CI of the Infection Fatality Rate (CFR(t)).

References

1. Li, Q. *et al.* Early transmission dynamics in wuhan, china, of novel coronavirus–infected pneumonia. *New England Journal of Medicine* (2020).
2. Wang, C. *et al.* Evolving epidemiology and impact of non-pharmaceutical interventions on the outbreak of coronavirus disease 2019 in wuhan, china. *MedRxiv* (2020).
3. Wang, D. *et al.* Clinical characteristics of 138 hospitalized patients with 2019 novel coronavirus–infected pneumonia in wuhan, china. *Jama* **323**, 1061–1069 (2020).
4. Zhou, F. *et al.* Clinical course and risk factors for mortality of adult inpatients with covid-19 in wuhan, china: a retrospective cohort study. *The lancet* (2020).
5. Coulson, T., Rohani, P. & Pascual, M. Skeletons, noise and population growth: the end of an old debate? *Trends in Ecology & Evolution* **19**, 359–364 (2004).
6. Bretó, C., He, D., Ionides, E. L., King, A. A. *et al.* Time series analysis via mechanistic models. *The Annals of Applied Statistics* **3**, 319–348 (2009).
7. Mode, C. J. & Sleeman, C. K. *Stochastic processes in epidemiology: HIV/AIDS, other infectious diseases, and computers* (World Scientific, 2000).
8. Lekone, P. E. & Finkenstädt, B. F. Statistical inference in a stochastic epidemic seir model with control intervention: Ebola as a case study. *Biometrics* **62**, 1170–1177 (2006).
9. Keeling, M. J. & Rohani, P. *Modeling infectious diseases in humans and animals* (Princeton University Press, 2011).
10. Jacquez, J. A. & O'Neill, P. Reproduction numbers and thresholds in stochastic epidemic models i. homogeneous populations. *Mathematical Biosciences* **107**, 161–186 (1991).
11. Diekmann, O., Heesterbeek, J. & Roberts, M. G. The construction of next-generation matrices for compartmental epidemic models. *Journal of the Royal Society Interface* **7**, 873–885 (2010).
12. Britton, T. Basic stochastic transmission models and their inference. *Handbook of Infectious Disease Data Analysis* 111 (2019).
13. Ridenhour, B., Kowalik, J. M. & Shay, D. K. Unraveling r0: Considerations for public health applications. *American journal of public health* **108**, S445–S454 (2018).
14. Liu, Y., Gayle, A. A., Wilder-Smith, A. & Rocklöv, J. The reproductive number of covid-19 is higher compared to sars coronavirus. *Journal of travel medicine* (2020).
15. Ridenhour, B., Kowalik, J. M. & Shay, D. K. Unraveling r 0: Considerations for public health applications. *American journal of public health* **108**, S445–S454 (2018).
16. Bertozzi, A. L., Franco, E., Mohler, G., Short, M. B. & Sledge, D. The challenges of modeling and forecasting the spread of covid-19. *arXiv preprint arXiv:2004.04741* (2020).
17. Oliver, N. *et al.* Mobile phone data for informing public health actions across the covid-19 pandemic life cycle (2020).
18. Unacast. The unacast social distancing scoreboard (2020). URL `https://unacast.com/post/the-unacast-social-distancing-scoreboard`.
19. Havers, F. P. *et al.* Seroprevalence of antibodies to sars-cov-2 in 10 sites in the united states, march 23-may 12, 2020. *JAMA Internal Medicine* (2020).
20. Los Angeles County Department of Public Health. La county daily covid-19 data dashboard (2020). URL `http://publichealth.lacounty.gov/media/coronavirus/data/index.htm`.
21. Los Angeles Times Data and Graphics Department. california-coronavirus-data github (2021). URL `https://github.com/datadesk/california-coronavirus-data`.
22. of Public Health, C. D. California department of public health open data portal (2021). URL `https://data.ca.gov/dataset/covid-19-hospital-data1`.
23. Jabot, F., Faure, T. & Dumoulin, N. Easy abc: performing efficient approximate b ayesian computation sampling schemes using r. *Methods in Ecology and Evolution* **4**, 684–687 (2013).
24. Marjoram, P., Molitor, J., Plagnol, V. & Tavaré, S. Markov chain monte carlo without likelihoods. *Proceedings of the National Academy of Sciences* **100**, 15324–15328 (2003).
25. Wegmann, D., Leuenberger, C. & Excoffier, L. Efficient approximate bayesian computation coupled with markov chain monte carlo without likelihood. *Genetics* **182**, 1207–1218 (2009).
26. Newcombe, P. J., Conti, D. V. & Richardson, S. Jam: a scalable bayesian framework for joint analysis of marginal snp effects. *Genetic epidemiology* **40**, 188–201 (2016).
27. Guan, W.-j. *et al.* Clinical characteristics of coronavirus disease 2019 in china. *New England journal of medicine* **382**,

1708–1720 (2020).

28. Petrilli, C. M. *et al.* Factors associated with hospitalization and critical illness among 4,103 patients with covid-19 disease in new york city. *MedRxiv* (2020).

29. Centers for Disease Control and Prevention (CDC), National Center for Health Statistics (NCHS). National Health and Nutrition Examination Survey Data (NHANES) (2017-2018). URL https://www.cdc.gov/nchs/nhanes/index.htm.

30. Los Angeles County Department of Public Health. Los angeles county health survey (LACHS) (2018). URL http://publichealth.lacounty.gov/ha/hasurveyintro.htm.

31. UCLA Center for Health Policy Research, C. A. .-., Los Angeles. California health information survey (2018). URL http://ask.chis.ucla.edu.

32. Los Angeles County Department of Public Health. Los angeles county case counts, crude rates by selected region (2020). URL https://github.com/AbigailHorn/COV2-LA/tree/master/data.

33. U.S. Census Bureau via R package tidycensus. Selected age groups, 2014-2018 american community survey 5-year estimates (2018). URL https://CRAN.R-project.org/package=tidycensus.

34. Strum, R. & Hattori, A. Morbid obesity rates continue to rise rapidly in the us. *International Journal of Obesity (London)* **37**, 889–891 (2013).
